# Optimization of YF17D-Vectored Zika Vaccine Production by Employing Small-Molecule Viral Sensitizers to Enhance Yields

**DOI:** 10.3390/vaccines13070757

**Published:** 2025-07-16

**Authors:** Sven Göbel, Tilia Zinnecker, Ingo Jordan, Volker Sandig, Andrea Vervoort, Jondavid de Jong, Jean-Simon Diallo, Peter Satzer, Manfred Satzer, Kai Dallmeier, Udo Reichl, Yvonne Genzel

**Affiliations:** 1 Bioprocess Engineering, Max Planck Institute for Dynamics of Complex Technical Systems, Sandtorstr. 1, 39106 Magdeburg, Germany; 2 ProBioGen AG, 13086 Berlin, Germany; 3 Virica Biotech, Ottawa, ON K1Y 2C5, Canada; 4 The Ottawa Hospital Research Institute, Ottawa, ON K1H 8L6, Canada; 5 Department of Biochemistry, Microbiology and Immunology, University of Ottawa, Ottawa, ON K1H 8M5, Canada; 6 p4b GmbH, 2120 Wolkersdorf, Austria; 7 KU Leuven Department of Microbiology, Immunology & Transplantation, Rega Institute, Molecular Vaccinology and Vaccine Discovery (MVVD), 3000 Leuven, Belgium; 8 Bioprocess Engineering, Otto-von-Guericke-University Magdeburg, Universitätsplatz 2, 39106 Magdeburg, Germany

**Keywords:** bioprocess engineering, vectored live-attenuated vaccines, viral sensitizers, small molecules, design of experiments, antiviral defense

## Abstract

**Background:** Modern viral vector production needs to consider process intensification for higher yields from smaller production volumes. However, innate antiviral immunity triggered in the producer cell may limit virus replication. While commonly used cell lines (e.g., Vero or E1A-immortalised cells) are already compromised in antiviral pathways, the redundancy of innate signaling complicates host cell optimization by genetic engineering. Small molecules that are hypothesized to target antiviral pathways (Viral Sensitizers, VSEs) added to the culture media offer a versatile alternative to genetic modifications to increase permissiveness and, thus, viral yields across multiple cell lines. **Methods:** To explore how the yield for a chimeric Zika vaccine candidate (YF-ZIK) could be further be increased in an intensified bioprocess, we used spin tubes or an Ambr15 high-throughput microbioreactor system as scale-down models to optimize the dosing for eight VSEs in three host cell lines (AGE1.CR.pIX, BHK-21, and HEK293-F) based on their tolerability. **Results:** Addition of VSEs to an already optimized infection process significantly increased infectious titers by up to sevenfold for all three cell lines tested. The development of multi-component VSE formulations using a design of experiments approach allowed further synergistic titer increases in AGE1.CR.pIX cells. Scale-up to 1 L stirred-tank bioreactors and 3D-printed mimics of 200 or 2000 L reactors resulted in up to threefold and eightfold increases, respectively. **Conclusions:** Addition of single VSEs or combinations thereof allowed a further increase in YF-ZIK titers beyond the yield of an already optimized, highly intensified process. The described approach validates the use of VSEs and can be instructive for optimizing other virus production processes.

## 1. Introduction

The yellow fever (YF) 17D vaccine is known for its exceptional efficacy and favorable safety profile, making it a promising viral vector for the development of live-attenuated recombinant vaccines [1,2]. Despite its small genome and limited coding capacity, YF17D can be genetically engineered to incorporate and express foreign antigens, enabling the design of chimeric or transgenic vaccine candidates [2]. One key advantage of YF17D over other viral vectors is its ability to provide dual immunity, offering protection against both YF and the target pathogen [2,3,4,5]. This may be particularly beneficial in regions where multiple infectious diseases co-circulate, as it could enhance public health impact while optimizing immunization efforts in areas with limited healthcare infrastructure. To ensure cost-effective, rapid, robust, and scalable manufacturing of viral vectors that can meet the growing global vaccine demand [6], outdated methodologies, such as egg-based production and use of adherent cell culture [7,8], need to be modernized and adapted to more efficient platforms. Several biological or engineering strategies have been employed in order to increase viral vector yields, including transition towards suspension cell culture [9,10,11], use of chemically defined media, optimized cultivation parameters, and process intensification strategies utilizing fed-batch [12] or perfusion cultures [13,14,15,16].

One often overlooked aspect in viral vector manufacturing is the impact of innate cellular antiviral defenses. Recent studies have shown that the cellular response to ectopic genetic material can activate antiviral pathways that reduce viral production efficiency [17,18,19]. While various strategies, such as RNA interference [20,21] or genomic knockout [22], have been explored to circumvent these cellular barriers, identification of specific targets for genetic engineering remains challenging due to the complexity of the innate antiviral network. Moreover, the process of single-cell cloning of recombinant cells and subsequent validation for consistency as required for regulatory approval is a time-consuming and costly process, especially if each new cell line needs to be fully characterized and produced under GMP conditions [23]. Small molecules, which make up 90% of pharmaceuticals [24], have shown promise in viral vector manufacturing due to their ability to target both extracellular (e.g., surface receptors) and intracellular components (e.g., kinases and transcription factors) of these antiviral pathways. For example, sodium butyrate, a histone deacetylase inhibitor, is commonly used to enhance lentiviral vector production or baculovirus transgene expression, though its effects have been inconsistent across different constructs [25,26]. More recent studies have explored small molecules such as nocodozale and M344 to improve AAV vector production, but none of these strategies specifically target innate antiviral defense pathways [27].

Another critical challenge in industrial viral vector manufacturing is process scale-up. Traditional upscaling routines often fail to produce consistent results in the transition from research scale to industrial production volumes. Underlying causes that contribute to this observation are heterogeneities of important process parameters in large mammalian cell bioreactors, a phenomenon that has been studied increasingly in recent years. Single-bioreactor or multi-bioreactor setups with oscillating pH or oxygen conditions are commonly used to evaluate the impact of pH and DO variations found in production-scale bioreactors [28,29,30,31]. While oscillating conditions are relatively easy to implement, they often lack representation, as they do not fully reflect the conditions at a large scale, considering that all cells are exposed to the applied conditions simultaneously [32]. Multicompartment systems comprising two or more interconnected bioreactors offer more representative models but are complex to set up. To ensure similar nutrient and gas gradients across scales [33], typical strategies focus on keeping one of three key parameters constant: volumetric power (P/V), tip speed of the stirrer blades, or mixing time. However, traditional vessels can only hold one of these settings constant, making process scale-up challenging. For example, maintaining constant P/V results in an increase in tip speed, shear force, and mixing time, while holding tip speed constant leads to a decrease in P/V and an increase in mixing time. Similarly, if mixing time is kept constant, both P/V and tip speed will increase during scale-up. By leveraging generative artificial intelligence (AI), novel bioreactor geometries can be designed and 3D-printed into perfect down-scale (pDS) reactors to achieve mixing times typically seen in larger-scale vessels without altering key parameters such as tip speed or P/V [34].

This study started from knowledge of a previous cell line screening for the high-yield production of YF-ZIK in suspension cell lines [9]. Based on this screening, in the current study, a low producer (BHK-21), a middle producer (AGE1.CR.pIX), and a high producer (HEK293-F) cell line were used to investigate whether and to what extent the addition of viral sensitizers (VSEs) has the potential to increase YF-ZIK yields. VSEs are derived from small-molecule compounds initially identified through high-throughput screening [35] that block innate antiviral responses. Many such molecules have been described in the literature, and they target a range of pathways that culminate in the blocking of antiviral responses [27,36,37,38,39,40]. Thus, identified VSEs were developed and selected for application in scalable virus biomanufacturing [35]. Starting from eight VSEs, we established working dose ranges based on tolerability studies and selected candidates that increased YF-ZIK titers compared to a control infection. Following dose optimizations in spin tubes or in a fully automated single-use Ambr15 microbioreactor system, we achieved significant fold-increases in all three host cell lines of up to sevenfold compared to control infections without VSEs. By combining multiple VSEs into a single formulation, we were able to further increase YF-ZIK titers, achieving synergistic increases up to 5.8-fold for AGE1.CR.pIX cells. Finally, the feasibility of using VSEs in a larger-scale manufacturing process was explored by scaling up to 1 L STRs and to 3D-printed 200 or 2000 L mimicked pDS reactors. Likewise, VSE addition significantly increased YF-ZIK titers by up to threefold and eightfold, respectively.

## 2. Materials and Methods

### 2.1. Cell Lines, Media, and Viral Seed Stock

Three suspension cell lines (AGE1.CR.pIX, BHK-21, and HEK293-F) were cultivated in baffled shake flasks (Corning, Corning, NY, USA; 50 mL working volume (wv)) or 50 mL vented Falcon spin tubes (Corning, USA, 30 mL wv) using a Multitron Pro incubator (Infors AG, Bottmingen, Switzerland, 50 mm orbital throw). AGE1.CR.pIX cells (ProBioGen, Germany) were grown in chemically defined CD-U7 medium (Xell/Sartorius, Bielefeld, Germany), supplemented with 2 mM alanine, 2 mM glutamine (Sigma Aldrich, St. Louis, MO, USA), and 10 ng/mL recombinant insulin growth factor (LONG-R3 IGF-I, Repligen, Waltham, MA, USA). BHK-21 cells (in-house adapted) were cultured in Protein Expression Medium (PEM, Gibco, Waltham, MA, USA) with 8 mM L-glutamine and 4 mM pyruvate (Sigma-Aldrich, USA). HEK293-F cells (in-house adapted, #R79007 ThermoFisher, Waltham, MA, USA) were maintained in chemically defined Dynamis^TM^ medium (Gibco, USA) without additional supplementation. BHK-21 and AGE1.CR.pIX cells were cultivated at 185 rpm, 37 °C, and 5% CO_2_, while HEK293-F cells were grown at 130 rpm, 37 °C, and 8% CO_2_. All suspension cultures were seeded at viable cell concentrations (VCCs) ranging from 5.0 to 8.0 × 10^5^ cells/mL and passaged twice a week. Adherent PS cells (RKI, Berlin, Germany) were cultivated in GMEM medium (ThermoFisher, Waltham, MA, USA) supplemented with LaB-M-peptone and 10% fetal calf serum (ThermoFisher, Waltham, MA, USA) at 37 °C and 5% CO_2_. The cells were seeded with an initial VCC of 3.0–5.0 × 10^5^ cells/mL in 50 mL and 200 mL using T175 cell culture flasks (Greiner, Frickenhausen, Germany) or 490 cm^2^ roller bottles (Greiner, Frickenhausen, Germany), respectively. Cell diameter, VCC, and percentage of viability were measured using an automated Vi-CELL XR cell counter (Beckman Coulter, Brea, CA, USA).

For infections, the previously described Vero E6-derived chimeric YF-ZIKprM/E virus (YF-ZIK) was used [9].

### 2.2. Tolerability Screening of Small Molecules in Spin Tubes

Initial working stocks of eight small molecules, referred to as VSEs, were prepared at 100 mM in either dimethyl sulfoxide (DMSO, 100% *v*/*v*) or sterile ultrapure water (Milli-Q, Millipore, Burlington, MA, USA). The VSEs used in this study were VS-A, VS-B, VS-C, VS-D, VS-E, VS-F, VS-G, and VS-H (Virica Biotech, Ottawa, ON, Canada). To determine a suitable working dose range (80–100% culture viability after 4–5 d of incubation), concentrations of 0.5–1000 µM (n = 2) were tested to evaluate tolerability profiles of the individual VSEs compared to PBS (control, n = 4). Working stocks were further diluted in the respective cell line medium and added to uninfected cells seeded at 2.0 × 10^6^ cells/mL in 50 mL vented Falcon spin tubes at 37 °C. All cells (AGE1.CR.pIX, BHK-21, and HEK293-F) were harvested 5 days post-addition, and VCC and viability percentages were measured.

### 2.3. Effect of VSEs on Virus Titers

To assess the impact of VSEs on virus production, low, medium, and high concentrations of each VSE (based on the established working dose range) were tested, and the virus yields were compared to control infections without VSEs. As before, cells (AGE1.CR.pIX, BHK-21, and HEK293-F) were seeded at 2.0 × 10^6^ cells/mL in 50 mL vented Falcon spin tubes (30 mL wv), with VSEs added at the respective concentrations. Cultures were subsequently infected with YF-ZIK at a multiplicity of infection (MOI) of 10^−2^ within 5 min after VSE addition. Spin tubes were sampled daily to measure the VCC, culture viability, and virus titer. Experiments were all performed at 37 °C. Scouting experiments were performed as single runs, as subsequent dose refinement was conducted iteratively through multiple rounds of narrowing the concentration range.

For AGE1.CR.pIX and HEK293-F cells, VSEs that increased infectious virus titers compared to control infections (n = 6) were selected for further testing across additional concentrations. A total of 4–6 concentrations per VSE were evaluated in biological replicates (n = 1 to n = 6), depending on the cell line and VSE.

For AGE1.CR.pIX, the three best-performing VSEs (VS-B, VS-F, and VS-G) were transferred to an Ambr15 system (Sartorius, Germany). Virus production was carried out as described previously [9]. Briefly, cells were seeded at 8.0 × 10^5^ cells/mL in 15 mL wv and cultivated at 37 °C and 800 rpm. Dissolved oxygen was maintained at 50% saturation by sparging an air–oxygen mixture, and pH was controlled at 7.2 by the addition of 1 M sodium bicarbonate (NaHCO_3_) or CO_2_ enrichment. For infections in the Ambr15 system, the wv was halved and replenished with fresh medium containing the VSEs (n = 3) or PBS (n = 9), resulting in a 1.7-fold dilution. Cells were then infected with YF-ZIK at an MOI of 10^−2^ within 15 min after VSE addition. After infection, cells were maintained at 37 °C, and vessels were sampled daily to measure VCC, offline pH, and virus titer.

### 2.4. Multi-Compound VSE Optimization Using Design of Experiments

Seeding, process control strategies, and infection procedures for all multi-compound experiments using AGE1.CR.pIX cells were carried out using the Ambr15 system and followed the same protocols as the single-compound Ambr15 experiments. To determine the optimal VSE concentrations for a two-compound approach (VS-G + VS-F and VS-G + VS-B; n = 2), a full factorial (FF) design of experiments (DoE) was employed. The effects of VSE concentrations on infectious virus titers were analyzed using two levels (L/H), as shown in Table S2, and they were compared to the control infection (n = 11). Factor levels were defined as low (L) and high concentrations (H) of the VSE. A complete factorial ANOVA was performed to evaluate the main effects and interactions between factors, with statistical significance set at α = 0.05. The effect size of factor interactions was measured according to the partial Eta-square from a 2 × 2 full-factorial ANOVA. Additionally, a response additivity approach [41,42] was used to calculate the combination index (CI) to investigate if the observed combination effect (E_AB_) was greater than the expected additive effect given by the sum of the individual effects (E_A_ + E_B_):

(1) $$CI=\frac{E_{A}+E_{B}}{E_{AB}}$$

A response additivity approach was selected to assess the interaction of VSE combinations, as it compares the effects from the combination with the expected effects from the single VSE addition, assuming an additive effect [42]. Despite the main drawback of assuming linear dose–effect curves for the VSEs when determining synergy, we selected the response additivity approach over more complex models such as Bliss independence [43] or Loewe additivity [44]. This decision was based on the limited size of our dataset and our aim to avoid assumptions of interaction mechanisms between VSE compounds.

In a second step, a reduced face-centered composite design (CCF) was used to optimize concentrations of three factors (VS-B, VS-F, VS-G; n = 1) across three levels (−1/0/1) to maximize infectious virus titers in a triple-dosing strategy (Table S3). Factor levels were defined as −1 and 1 for the low and high concentrations, respectively, and 0 for the center-point level. Center points were conducted in quintuplicates (n = 5) to assess model reproducibility and experimental variability. Furthermore, a quadruple combination strategy involving VS-B, VS-F, VS-G, and VS-H was evaluated at three levels (−1/0/1; Table S3). Experimental design and analysis of the results were performed using the MODDE 13 software (Sartorius, Umea, Sweden).

### 2.5. Generation of 3D-Printed Down-Scaled Reactors

Small-scale (1 L benchtop) reactors with similar mixing characteristics to those of large-scale bioreactors were generated according to WO2024133134 [34], which provides an algorithm that generates novel internal geometries within a bioreactor vessel using appropriate computational fluid dynamics (CFD) for the prediction of mixing kinetics. A digital twin of a large-scale reactor representing 200 and 2000 L as targets was generated, and the mixing behavior was simulated using the M-Star CFD simulation software v3.9.54 (Boston, MA, USA). Determination of mixing time and mixing curve was performed by using the digital twin by tracer injection at the feed addition point and recording the resulting homogeneity [45]. The total required mixing time was defined as the time interval required to reach 95% homogeneity of the tracer distribution within the simulated digital twin. Additionally, the local mixing time distribution was determined using the digital twin and a custom-made tool according to Fitschen et al. [45]. The results from the two models for determining mixing behavior were used as target values and objective function for an evolutionary algorithm. The algorithm introduces random changes into the internal geometry of the bioreactor and selects the best performing variant according to the objective function. The best variant was then again randomly modified, and this generational approach was repeated until the internal geometry of the mixing behavior generated by the small-scale reactor was similar to the large-scale target reactor. Once similar mixing behaviors to that of the target reactor (200 L or 2000 L) were achieved, the 3D model was exported, and the mimicked reactor was manufactured using 3D printing. The reactors were then contract manufactured in accordance with ISO 1348:2016 (International Organization for Standardization, “Medical Devices—Quality Management Systems—Requirements for Regulatory Purposes,” ISO 13485:2016, 2016) guidelines to produce medically certified parts using laser-sintering 3D-printers and Nylon PA12 as materials (Oceanz, Ede, The Netherlands).

### 2.6. Scale-Up to 1 L Benchtop Reactors and Evaluation of 3D-Printed Down-Scaled Reactors

The best multi-compound combinations of VSEs in AGE1.CR.pIX cells (VS-F + VS-G) and the single-compound addition in HEK293-F cells (VS-C) were scaled up to 1 L stirred DASGIP bioreactors (STR; Eppendorf AG, Hamburg, Germany). Previously established YF-ZIK batch production protocols were followed [9]. Briefly, cells were seeded at 8.0 × 10^5^ cells/mL with an initial wv of 350 mL. During cell growth, the culture temperature was maintained at 37 °C, and the pH value was controlled at 7.2 for both cell lines. For infection, the wv was increased to 700 mL through the addition of 350 mL of fresh medium containing the respective VSEs (n = 2) or PBS (n = 3) to achieve a 1:2 dilution. Cells were infected with YF-ZIK at an MOI of 10^−2^ within 5 min after VSE addition and the cultivation temperature was lowered to 33 °C post infection.

In a final step, the scale-up behavior and impact of mixing with addition of VSEs to mimicked 200 and 2000 L Xcellerex bioreactors (Cytiva, Marlborough, MA, USA) was investigated using the generated 1 L 3D-printed down-scaled reactors (pDS reactors) [34]. pDSs were also generated to fit into the previously used Eppendorf 1 L DASGIP system without changing the tip speed or power input. For this, the head-space set-up and cultivation and infection conditions were identical to those of the previous glass Eppendorf reactors. In total, two vessels with different mixing times, one with 49.9 s (equivalent to the 200 L Xcellerex) and one with 94 s (equivalent to the 2000 L Xcellerex), were investigated and compared to traditional 1 L glass vessels and a 3D-printed polymer vessel as a 3D-printed reference.

### 2.7. Plaque Assay and Real-Time Quantitative PCR

Infectious YF-ZIK titers were determined using a plaque assay with a coefficient of variance of 25% (±0.15 log), as previously described [9]. RNA extractions from cell culture supernatants were performed using the NucleoSpin RNA Virus Kit (Macherey-Nagel, Germany) according to the manufacturer’s instructions. YF-ZIK RNA copy numbers were quantified by qRT-PCR using previously published primers [14,46] and a probe targeting the ZIKV E protein (Table S1). Reactions were performed with One-Step TaqMan RT-PCR Master Mix reagents (Qiagen, Germantown, MD, USA) on a Rotor-Gene Q real-time PCR cycler (Qiagen, USA). The master mix (10 μL total volume) was prepared according to the manufacturer’s recommendations. Using a pipetting robot, 2 μL of RNA sample was added to 8 μL of the master mix in a RotorDisc 100 (Qiagen, USA) with an accuracy of 5%. Reverse transcription was carried out at 45 °C for 10 min, followed by denaturation at 95 °C for 5 min. Amplification consisted of 45 cycles of 95 °C for 5 s and 60 °C for 30 s. Melting curve analysis was performed from 65 °C to 90 °C. Based on the amplicon fragment length (77 bp), the average nucleotide mass (340 Da/bp), and RNA copy numbers from the standard dilution series, the total viral RNA (vRNA) copy number in the samples was calculated [47].

### 2.8. Calculations

Fold-increases were calculated using the following equation:

(2) $$Fold\;increase=\frac{c_{Vir,max,VSE}}{C_{vir,max,control}}$$

Here, c_vir,max,VSE_ represents the maximum infectious virus titer reached after VSE addition (PFU/mL), and c_vir,max,control_ represents the maximum infectious virus titer reached after PBS addition (PFU/mL).

### 2.9. Statistical Analysis

All statistical evaluations were carried out using GraphPad Prism V9 (GraphPad Software, Boston, MA, USA) or MODDE13 (Sartorius, Umea, Sweden). The data are presented as mean values ± standard deviation (STD). Statistical significance was assessed using a one-way ANOVA followed by Dunnett’s multiple-comparison test with respect to the control. Statistical differences between groups are denoted by *p*-values < 0.05: * *p*-values  <  0.05, ** *p*-values  <  0.01, *** *p*-values  <  0.001, **** *p*-values  <  0.0001.

### 2.10. Bioprocess Cost Modeling

BioSolve Process^TM^ economic modeling software v8.3 (Biopharm Services Ltd., Chesham, UK) was used for cost modeling. The software’s pre-loaded generic suspension cell-based vaccine model (named “Vaccine Typical”) was utilized with the default cost database and production parameters. The model accounted for major unit operations of a large-scale cell-based vaccine production process, including a seed train, fed-batch bioreactor production step, and downstream purification consisting of multiple chromatography and filtration steps. Three large-scale production process scenarios were simulated: a baseline production at a 2000 L scale and a conservative threefold increase in yield at 1000 L and 2000 L scales, respectively. To assess the economic impact of increasing the upstream viral yield through VSE addition, the resulting manufacturing costs per dose in all three scenarios were compared.

## 3. Results

### 3.1. Evaluation of the Tolerability of VSEs in Host Cell Lines

Based on previous results, we selected three host cell lines as substrates that differed significantly in origin and virus productivity [9]. HEK293-F cells (human) exhibited high YF-ZIK productivity and were classified as high producers. AGE1.CR.pIX cells (avian) supported YF-ZIK growth to moderate titers. In contrast, BHK-21 cells (rodent) were poor producers with low virus titers. VSEs interfere with host cell metabolism, signaling, and viability. Considering the potential impact of factors such as cell substrate and culture media on VSE tolerance, a comprehensive screening was conducted to assess the tolerability of a panel of eight VSEs across the three host cell lines. Suitable dosing ranges were defined based on culture viability, with the objective of maintaining 80–100% target viability at day 5 post-addition, corresponding to the optimal virus harvest time point [9]. Mimicking a YF-ZIK batch production process, VSEs were added to non-infected cells seeded at 2.0 × 10^6^ cells/mL at concentrations ranging from 0.5 to 1000 µM and compared to a PBS control. As expected, the host cell lines exhibited varying tolerances to the respective VSEs, and higher VSE concentrations led to significantly reduced VCCs. The tolerability ranges of each VSE are shown in Figures S1–S3. Based on these results, the maximum input doses for a first round of infection experiments were defined.

### 3.2. Effect of VSEs on YF-ZIK Titers

**Figure 1:**
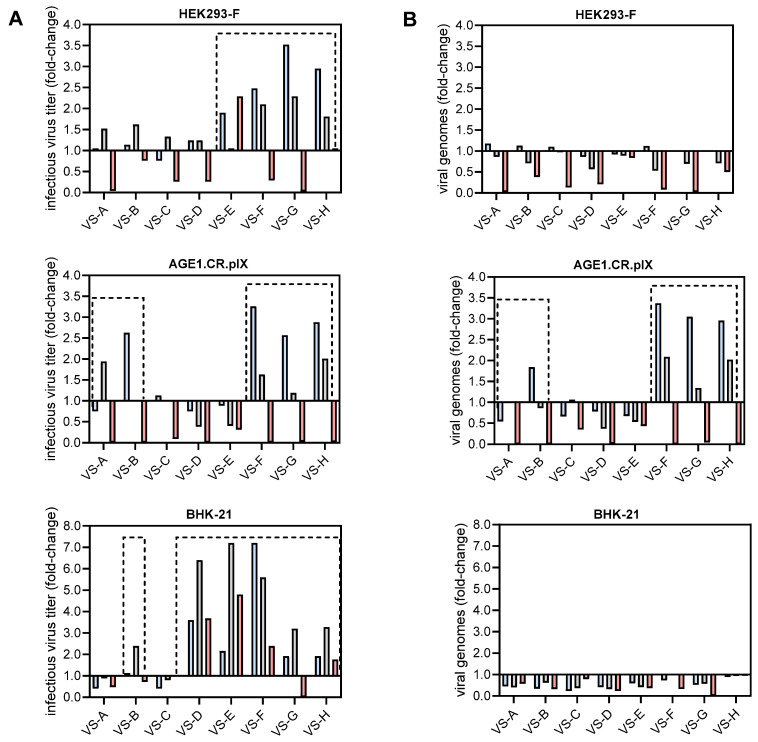
Initial screening of eight VSEs to increase infectious YF-ZIK titers in three cell lines. Low (blue), medium (gray), and high (red) concentrations of each VSE were used. (**A**) Maximum infectious virus titers (PFU/mL) are shown as fold-changes relative to YF-ZIK-producing cells without VSEs. (**B**) Fold-changes in maximum total viral genomes measured using qRT-PCR. Promising VSEs are highlighted with dashed boxes. Values represent single experiments carried out in spin tubes (n = 1).

As a first step of optimization, the impact of the eight VSEs on YF-ZIK production was evaluated for all three cell lines. Using the previously established working dose ranges for each VSE and cell substrate, low, medium, and high concentrations were tested in spin tubes, and the resulting maximum virus titers were compared to a control infection (water instead of VSE). The high concentration was defined as the intersection point of the interpolated dose–response curve with a horizontal line at 80% viability (Figures S1–S3). Changes in maximum infectious virus titers or total viral genomes (vg) were expressed as fold-changes, where values > 1 indicate an increase and values < 1 a decrease relative to the control infection without VSEs (Figure 1).

For all cell lines, concentration-dependent effects of the VSEs on maximum infectious virus titers and total viral genomes (vgs) were observed (Figure 1A,B). In HEK293-F cells, certain concentrations of VS-E, VS-F, VS-G, and VS-H resulted in an increase in infectious virus titers, with fold-changes exceeding 2.5 compared to the control. In contrast, VSEs VS-A to VS-D showed limited or inhibitory effects on YF-ZIK production. For AGE1.CR.pIX cells, low or medium concentrations of VS-A, VS-B, VS-F, VS-G, and VS-H resulted in up to threefold increases in infectious virus titers compared to the control infection. In general, the addition of VSEs to BHK-21 cells led to the highest increases in infectious virus titers, with low or medium concentrations of VS-E and VS-F resulting in up to sevenfold higher infectious virus titers (Figure 1A).

Interestingly, only for AGE1.CR.pIX cells, the fold-changes in vgs and infectious virus titers were consistent. Addition of VSEs to both HEK293-F and BHK-21 cells resulted in lower total vgs compared to the control infection, suggesting a higher proportion of infectious virus particles despite the lower YF-ZIK production (Figure 1B).

Based on this initial screening, four VSEs per cell substrate were selected for further investigations. Despite the high improvements for BHK-21 cells, the maximum infectious virus titers were still >3 log lower than those in AGE1.CR.pIX and HEK293-F cells (Figure 6). Therefore, BHK-21 cells were excluded from all subsequent experiments.

**Figure 2:**
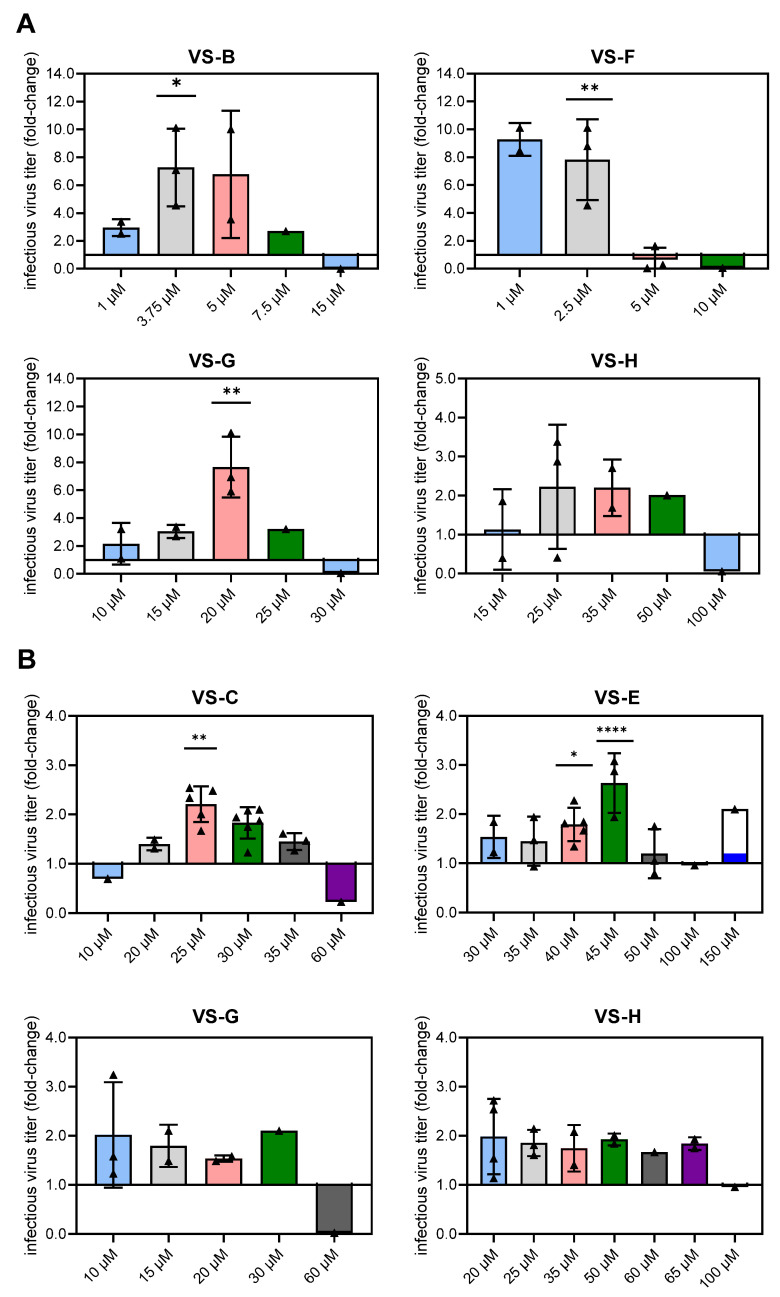
Dose-dependent effect of selected VSEs in AGE1.CR.pIX (**A**) and HEK293-F (**B**) cells to increase YF-ZIK titers. Maximum infectious virus titers (PFU/mL) are shown as fold-changes relative to YF-ZIK-producing cells without VSEs. Error bars represent the mean ± STD of biological replicates (n = 1–6 for VSEs and n = 6 for the control) carried out in spin tubes. Normality was assessed using the Shapiro–Wilk test. Data (n ≥ 3) were analyzed using one-way ANOVA, followed by Dennett’s comparison test, for comparison with the control. Significant differences are indicated by asterisks (* *p* < 0.05; ** *p* < 0.01; **** *p* < 0.0001).

Assuming a bell-shaped curve for VSE concentration and the effect on virus production, additional experiments were performed to determine the optimal VSE concentrations more precisely in an iterative manner. VS-B, VS-F, VS-G, and VS-H were selected for AGE1.CR.pIX cells, and VS-C, VS-E, VS-G, and VS-H were selected for HEK293-F (Figure 2). As anticipated, the relationship between VSE concentration and infectious virus titer enhancement exhibited a Gaussian rather than linear relationship for certain VSEs (e.g., VS-B and VS-G in AGE1.CR.pIX; VS-C and VS-E in HEK293-F; Figure 2), with each VSE demonstrating a distinct optimal concentration. While most VSE concentrations led to an increase in infectious virus titers in AGE1.CR.pIX cells, only the addition of 3.75 µM VS-B (*p* = 0.037), 2.5 µM VS-F (*p* = 0.004), and 20 µM VS-G (*p* = 0.002) resulted in statistically significant improvements, achieving an up to eightfold increase compared to the control (Figure 2A). Similarly, in HEK293-F cells, VS-G and VS-H generally enhanced YF-ZIK production across multiple concentrations, but only the addition of 25 µM VS-C (*p* = 0.005), 40 µM VS-E (*p* = 0.024), and 45 µM VS-E (*p* < 0.0001) yielded significant increases of up to threefold relative to the control (Figure 2B). As previously observed (Figure 1B), the fold-changes in vgs and infectious virus titers exhibited similar trends for both AGE1.CR.pIX and HEK293-F cells, with the fold-changes in vgs generally being smaller than and not as pronounced as those in infectious virus titers (Figure S4), suggesting that the addition of VSEs mainly enhanced the production of mature infectious viral particles. As YF-ZIK is a live-attenuated vaccine, we prioritized the measurement of infectious virus titers for all subsequent experiments as the parameter most relevant to vaccine efficacy and potency.

**Figure 3:**
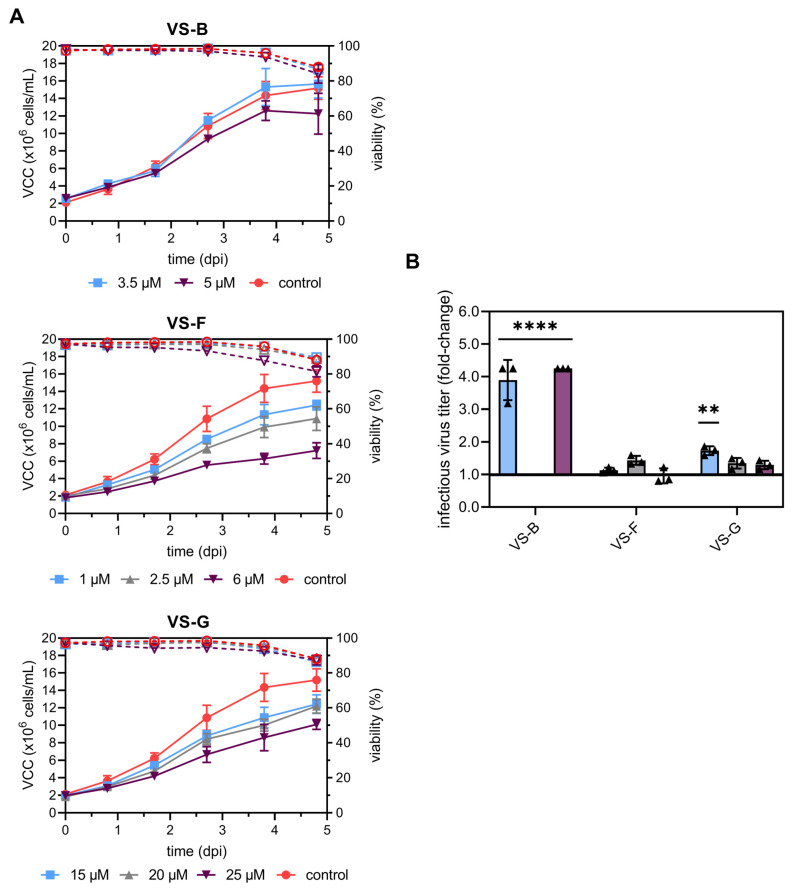
Evaluation of selected VSE concentrations for YF-ZIK production in AGE1.CR.pIX cells using an Ambr15 system. Cell growth and fold-changes in infectious virus titers (PFU/mL) using an Ambr15 system. (**A**) VCC (full symbols) and cell viability (empty symbols) are shown for AGE1.CR.pIX cells grouped by VSE. Control infections (sterile water instead of VSEs) are shown as red circles. (**B**) Maximum infectious virus titers are shown as fold-changes relative to the control infections. Error bars represent the mean ± STD of biological replicates (n = 3 for VSEs and n = 11 for the control). Normality was assessed using the Shapiro–Wilk test. Data (n ≥ 3) were analyzed using one-way ANOVA, followed by Dennett’s comparison test, for comparison with the control. Significant differences are indicated with asterisks (** *p* < 0.01; **** *p* < 0.0001).

As a final step in process optimization, we chose AGE1.CR.pIX cells, the moderate but GMP-compliant producer, as the cell substrate. We then transitioned virus production to an automated high-throughput Ambr15 bioreactor system to ensure controlled and reproducible conditions. Under these controlled process conditions, VS-B, VS-F, and VS-G demonstrated significant increases in infectious virus titers in AGE1.CR.pIX cells and were reevaluated at the previously identified optimal concentrations, including concentrations immediately below and above the previously defined optimum. A robust and reproducible YF-ZIK production was achieved with consistent trends and lower standard deviations in VCC and infectious virus titers across all tested conditions and replicates (n = 3 for VSEs and n = 11 for the control). Maximum VCCs of 15.3 ± 1.1 × 10^6^ cells/mL were reached at day 5 pi for the control infection. A concentration-dependent inhibitory effect of VSEs on VCC was observed (Figure 3A). The most pronounced reduction was a two-fold decrease in maximum VCC compared to the control at 5 d post-infection (pi) and was observed with VS-F (6 µM) and VS-G (25 µM). For VS-B, a similar yet less pronounced decrease in maximum VCCs was observed with increasing concentrations. Despite these effects, the culture viability across all compounds and concentrations remained comparable to the control, maintaining >80% viability at 5 dpi. Compared to spin-tube infections, the fold-changes of infectious virus titers were lower in the Ambr15 system (Figure 3B), most likely due to differences in hydrodynamics, mixing, and process controls, but the standard deviation between replicates was notably reduced. The addition of 3.5 µM and 5 µM VS-B (*p* < 0.0001), as well as 15 µM VS-G (*p* = 0.006), led to significant increases, with VS-B achieving an up to fourfold improvement and VS-G a twofold improvement relative to the control (Figure 3B). For VS-F, no significant increase was observed at any concentration tested.

### 3.3. Multi-Compound VSE Optimization Using DoE

**Figure 4:**
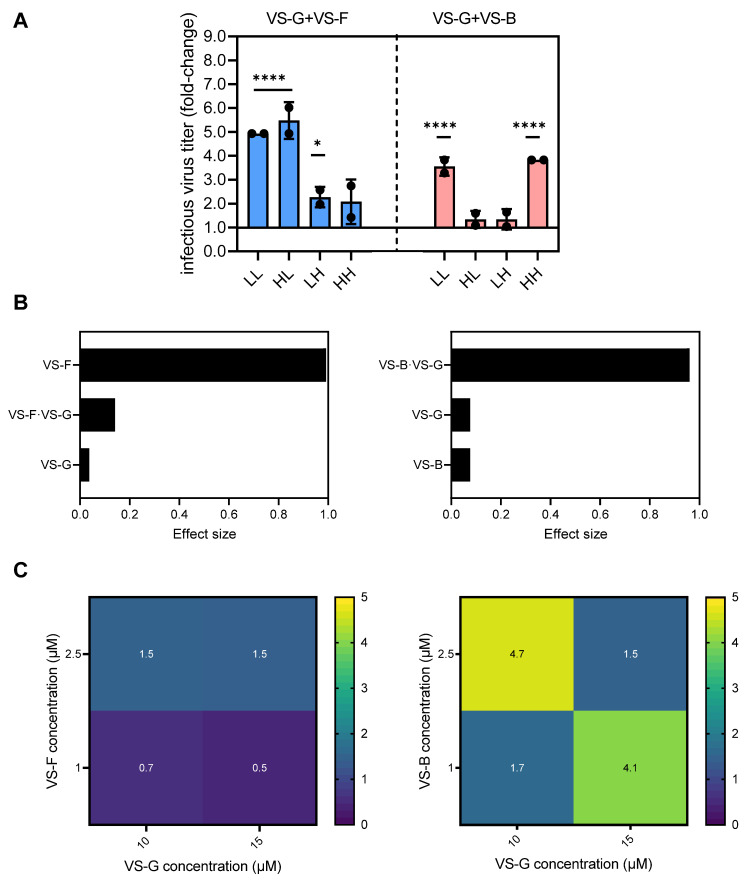
Evaluation of synergistic and antagonistic effects of combinations of two VSEs in AGE1.CR.pIX cells using a full-factorial (FF) DoE design. (**A**) Maximum infectious virus titers (PFU/mL) are shown as fold-changes relative to the control infections (n = 13). Error bars represent the mean ± STD of biological duplicates. Significant differences are indicated with asterisks (* *p* < 0.05; **** *p* < 0.0001). (**B**) Effect size of factors and factor interactions measured using the partial Eta-square from a 2 × 2 FF ANOVA. The statistical significance was set at 0.05. (**C**) Synergy plot showing a combination index (CI) analysis according to the response additivity approach. CI values >1 indicate antagonism, =1 indicate additivity, and <1 indicate synergy. All experiments were carried out in the Ambr15 system.

To assess whether specific combinations of two VSEs could act synergistically to further enhance YF-ZIK production in AGE1.CR.pIX cells, we employed an FF DoE approach. We selected the optimal concentrations (referred to as High, H) identified in the Ambr15 run (2.5 µM for VS-F, 3.5 µM for VS-B, and 15 µM for VS-G), along with one concentration below the optimum (1 µM for VS-F, 1 µM for VS-B, and 10 µM for VS-G; referred to as Low, L), to account for the anticipated higher cytotoxicity when combining two VSEs compared to using a single VSE (Figure 4). In this stage, two factors and their one possible interaction were evaluated. Combination of VS-F and VS-G resulted in higher fold-increases compared to individual addition, up to 5.4-fold for LL and HL (*p* > 0.0001) compared to 1.4- and 2-fold for single addition, respectively (Figure 3B and Figure 4A). On the other hand, combining VS-B and VS-G yielded similar fold-increases to that for single addition (4-fold, Figure 3B and Figure 4A). A two-way factorial ANOVA was performed to evaluate the effects of VS-G + VS-B and VS-G + VS-F on the response variable. For VS-G and VS-F, only the main effect of VS-F was significant (F(1,4) = 446, *p* = 0.0026, η^2^ = 0.991), accounting for 99.1% of the variance in the response (Figure 4B). Both the main effect of VS-G (F(1,4) = 0.15, *p* = 0.72, η^2^ = 0.04) and their interaction (F(1,4) = 0.66, *p* = 0.46, η^2^ = 0.14) were not significant. For VS-G and VS-B, neither main effect significantly influenced the response. However, their interaction was significant (F(1,4) = 97, *p* = 0.0006, η^2^ = 0.96), explaining approximately 96% of the variance in the response, overshadowing their individual contributions (Figure 4B). To determine whether the combination of VSEs produced a synergistic effect, we calculated the CI using the response additivity approach. In this framework, CI values > 1 indicate antagonism, those equal to 1 indicate additivity, and those <1 indicate synergy. The combination of VS-F and VS-G exhibited a synergistic effect, particularly at low concentrations of VS-F (Figure 4C). The enhanced overall response when combined may suggest the action of these VSEs on different cellular pathways. In contrast, all combinations of VS-B and VS-G resulted in antagonistic effects, with the most pronounced antagonism being observed at LH/HL concentrations (Figure 4C).

**Figure 5:**
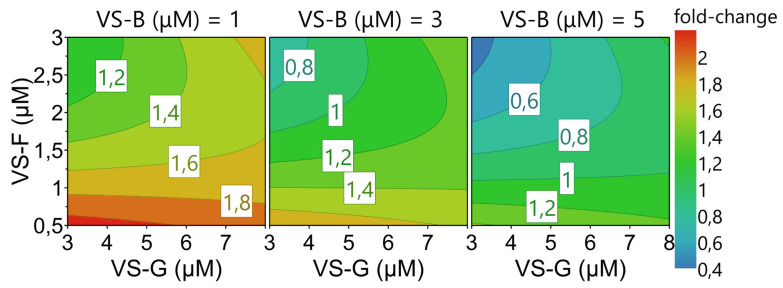
Four-dimensional response contour plot of a CCF DoE design investigating triple-VSE combinations in AGE1.CR.pIX cells using an Ambr15 system. Maximum infectious virus titers (PFU/mL) are shown as fold-changes relative to the control infections (n = 15). The influence of the respective VSE concentration on the fold-increase responses is shown.

Building on the observation that the combination of two VSEs, VS-G and VS-F, synergistically enhanced infectious YF-ZIK titers in AGE1.CR.pIX cells compared to their individual additions, a reduced CCF design was employed to explore whether infectious virus titers could be further improved using a triple-dosing strategy. A total of 21 experimental conditions, including five center-point replicates to ensure robust data collection, were evaluated for VS-B, VS-F, and VS-G. Multiple linear regression was applied to model the collected data, with iterative optimization achieved by removing insignificant coefficients unless they were linked to a significant coefficient (*p* < 0.05). Among the variables, VS-F and VS-B were identified as significant drivers of process outcomes (Figure S5). The model demonstrated satisfactory performance, with fit values (R^2^) of 0.61, predictability values (Q^2^) of 0.43, model validity values of 0.59, and reproducibility values of 0.74 for fold-changes in infectious virus titer responses (Figure S6). To visualize the model results, response contour plots were generated, depicting fold-increases in infectious virus titers relative to control infections across the VSE concentration ranges (Figure 5). The highest fold-increases, approximately twofold, were observed at low concentrations of all VSEs. However, these increases were notably lower compared to the effects observed with double or single additions of the respective VSEs (Figure 3B and Figure 4A). This observation was further validated through a second reduced CCF design, which investigated the addition of four VSEs (VS-B, VS-F, VS-G, and VS-H). In most experimental conditions, significant cell death occurred within 24 h following the addition of all four VSEs. This may indicate that the benefits of VSEs are lost when more than two are combined due to reduced tolerability.

**Figure 6:**
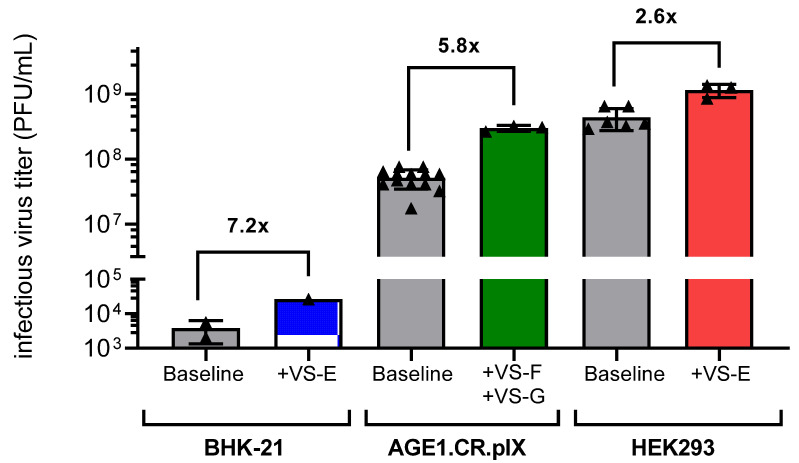
Maximum infectious YF-ZIK titers across various cell lines and production formats with and without VSE addition. BHK-21 and HEK293-F cells were cultivated in spin tubes, and AGE1.CR.pIX cells were cultivated in the Ambr15 system. For BHK-21 cells, the addition of 25 µM VS-E (n = 1) was compared to the control infection (n = 2). For AGE1.CR.pIX cells, the addition of 1 µM VS-F and 15 µM VS-G (n = 3) was compared to the control infection (n = 15). For HEK293-F cells, the addition of 45 µM VS-E (n = 3) was compared to the control infection (n = 6). Error bars represent the mean ± STD of replicates.

In summary, though most combinations resulted in antagonistic responses, selected combinations of VSEs could synergistically enhance infectious YF-ZIK titers in AGE1.CR.pIX cells compared to single additions. Combinations of more than two VSEs provided no additional benefits and resulted in antagonistic responses or cytotoxicity. An overview of the best conditions identified in spin tubes for BHK-21 and HEK293-F, as well as AGE1.CR.pIX in the Ambr15 system, is shown in Figure 6. For all three cell lines, addition of single or double VSEs resulted in significant increases of infectious virus titers (2.6−7.2 fold), without adding additional complexity to the production process.

### 3.4. Scale-Up to a Laboratory-Scale Stirred Tank Bioreactor

To assess the scalability of YF-ZIK production improvements achieved with VSEs optimized at a small scale (15 mL vessels), pilot runs were carried out in 1 L benchtop STRs analogous to our previously optimized conditions [9]. These experiments evaluated the combined addition of 1 µM VS-F and 15 µM VS-G to AGE1.CR.pIX cells, as well as the single addition of 25 µM VS-C to HEK293-F cells. While previous results showed that VS-E yielded higher infectious virus titers compared to VS-C, VS-C was selected for HEK293-F cells due to its more consistent performance over a broader concentration range, particularly at concentrations slightly below or above the identified optimum (Figure 2B).

**Figure 7:**
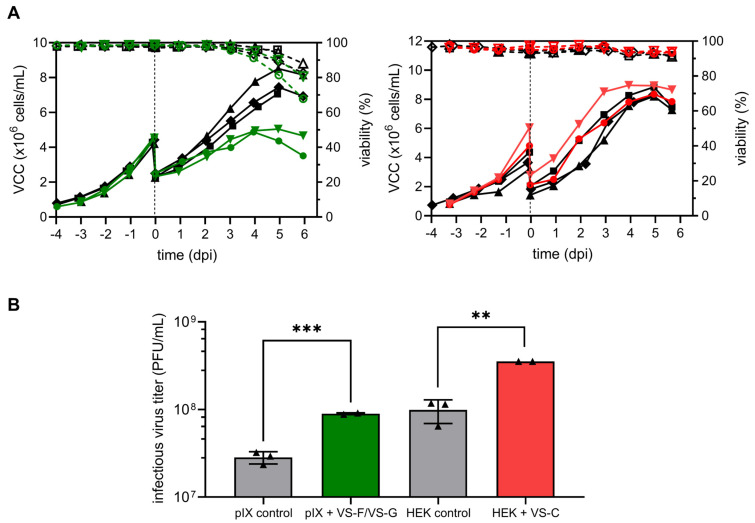
YF-ZIK production in benchtop 1 L STRs in batch mode using AGE1.CR.pIX (left, green) and HEK293-F cells (right, red) with or without VSE addition. VSEs (colored) or PBS (black) were added to the cells prior to infection. Replicates are represented by different symbols (controls: triangle, diamond, square; VSE addition: circle and downwards triangle); 1 µM VS-F and 15 µM VS-G were added to AGE1.CR.pIX cells, and 25 µM VS-C was added to HEK293-F cells. At the time of infection, cells were diluted with fresh medium at a ratio of 1:2, and the temperature was reduced to 33 °C. (**A**) VCC (full symbols) and culture viability (empty symbols). Dashed lines indicate the time of infection. (**B**) Maximum infectious virus titers determined with a plaque assay. Error bars represent the mean ± STD of biological replicates (n = 2 for VSEs and n = 3 for the control). Significant differences are indicated with asterisks (** *p* < 0.01; *** *p* < 0.001).

Both HEK293-F and AGE1.CR.pIX cells were seeded at 0.8 × 10^6^ cells/mL and cultivated at 37 °C until a VCC of about 4.0 × 10^6^ cells/mL (Figure 7A). Cells were diluted 1:2 with medium, and the temperature was shifted to 33 °C. After addition of the VSEs and/or infection, AGE1.CR.pIX cells continued to grow until 4–5 days pi. However, for both reactors containing VSEs, the maximum VCCs before onset of cell lysis (VCC_max_ 5.0 ± 0.1 × 10^6^ cells/mL) were lower compared to the control (VCC_max_ 7.7 ± 0.7 × 10^6^ cells/mL). For HEK293-F cells, there was no noticeable difference in VCC_max_ or culture viability pi between VSE addition (VCC_max_ 8.7 ± 0.4 × 10^6^ cells/mL) or the control (VCC_max_ 8.5 ± 0.4 × 10^6^ cells/mL) (Figure 7A). Although the VCC_max_ values for both the AGE1.CR.pIX control and VSE addition were twofold lower in the STR compared to the Ambr15 system, the maximum infectious virus titers of the control infections were similar for both (2.8 ± 0.4 × 10^7^ PFU/mL and 3.3 ± 1.7 × 10^7^ PFU/mL, respectively). Addition of VS-F and VS-G resulted in a significant increase (*p* = 0.0004) in the maximum infectious titer, yielding a 3.2-fold increase compared to the control infection (Figure 7B).

Cell growth pi in STR cultivations was comparable to that in spin tubes for HEK293-F cells for both the control and VSE addition. Maximum VCCs of 8.8 ± 0.4 × 10^6^ cells/mL and 8.3 ± 0.8 × 10^6^ cells/mL, respectively, were obtained despite the temperature reduction in the STR. Importantly, the addition of VS-C resulted in a significant 3.5-fold increase (*p* = 0.002) in the maximum infectious titer compared to the control infection.

Daily samples from the HEK293-F 1 L STR were analyzed for residual VS-C through liquid chromatography mass spectrometry (LC-MS) (methods and data provided in Supplementary File S1). Residual VS-C amounts from the treatment with 25 µM at the time of infection were already below the assay detection limit of 0.5 µM at day 1 pi (Supplementary File S1). This level of depletion suggests that VS-C is rapidly metabolized by HEK293-F cells under these conditions, drastically facilitating the downstream steps required to control residual VSE levels in a final drug product.

### 3.5. Mimicking Large-Scale Production Conditions in Small-Scale 3D-Printed Reactors

During the scale-up of biopharmaceutical production processes, unpredictable performance losses can arise due to gradients and heterogeneities [48,49,50]. Such effects are especially concerning if small molecules such as chemical inducers or enhancers for improved productivity, such as the VSEs used here, are supplemented at a desired cell concentration to start the production phase. Extended mixing times in large-scale bioreactors can furthermore create oxygen- and nutrient-depleted regions due to concentration gradients [49]. Consequently, cells undergo transient hypoxia and fluctuating nutrient availability, which can also negatively impact virus production.

**Figure 8:**
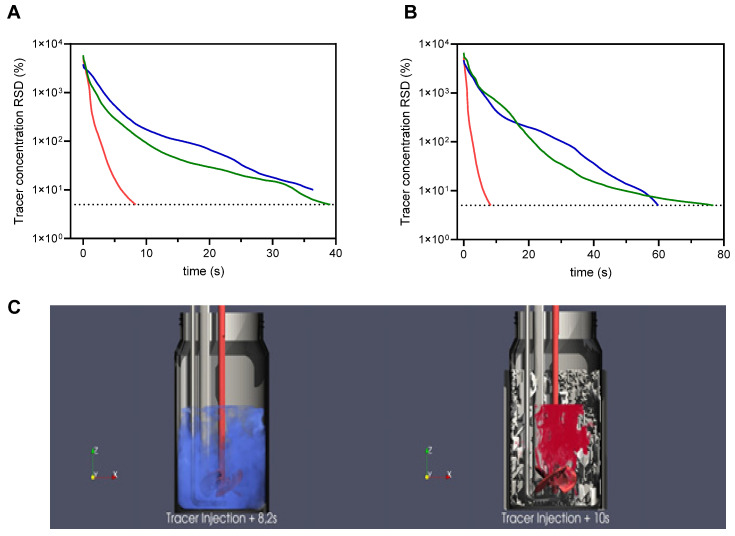
Simulated mixing curves for 200 L (**A**) and 2000 L (**B**) with the small-scale plain vessel without a structure (red), the target reactor (either 200 or 2000 L, blue), and the corresponding small-scale model including a structure (green). The dotted line shows the 95% threshold of the relative standard deviation of the scalar over the entire domain (RSD), representing homogenous mixing. (**C**) The structure of the 2000 L surrogate pDS reactor (right) compared to the small-scale plain vessel without a structure (left) and the tracer distribution after 10 s (red) and 8.2 s (blue), respectively. Depicted in red are the shaft and stirrer blade of the used stirrer at the respective stirrer height.

To mitigate performance losses, reliable tools are needed to analyze, predict, and ultimately prevent inconsistencies between laboratory- and commercial-scale production [48,50]. Replicating large-scale mixing behavior in small-scale bioreactors makes the use of internal structures or the use of two-vessel systems necessary [28,51]. Randomly generated structures can achieve this without generating hard boundaries within the bioreactor but result in complex geometries that need 3D-printing to be manufactured. To evaluate possible mixing issues with VSE additions, target reactors were defined as 200 L (reactor A) and 2000 L (reactor B) and generated to closely mimic the mixing curve of the respective single-use Xcellerex systems (see Section 2). CFD simulations were accelerated using single-phase simulations without the addition of sparging. Mixing curves for the original plain vessel, the 200 L and 2000 L targets, and the resulting pDS reactors are shown in Figure 8A,B alongside renders of the resulting complex structures with tracer distribution after approximately 10 s (Figure 8C). The mixing curves of the large-scale bioreactors were closely modeled with the structures generated in the pDS reactors and showed significant differences from the plain reactor without an internal structure.

**Figure 9:**
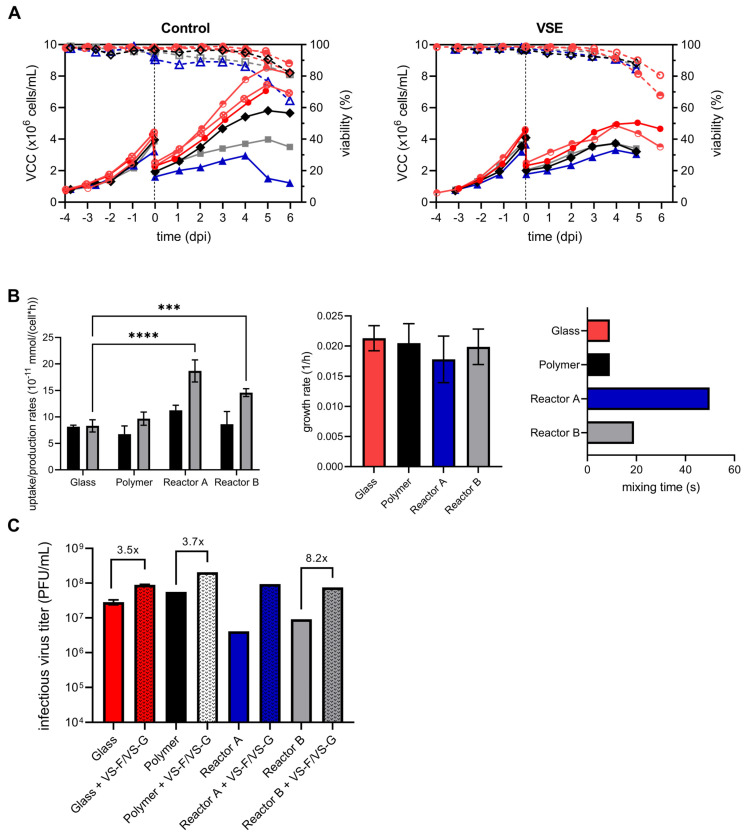
Comparison between plain glass (red), polymer 3D-printed (black), and 3D-printed pDS reactor A (blue) or pDS reactor B (gray) for YF-ZIK production using AGE1.CR.pIX cells with or without the addition of VSEs. Prior to infection, cells were diluted at a ratio of 1:2 with fresh medium, the temperature was reduced to 33 °C, and 1 µM VS-F and 15 µM VS-G or PBS were added to the cells. (**A**) VCC (full symbols) and culture viability (empty symbols). Dashed lines indicate the time of infection. (DO control failure in reactor A without VSE at 4 dpi.) (**B**) Glucose (black) and lactate (gray) uptake/production rates (left) and cell-specific growth rates (middle) prior to infection and/or VSE addition. Simulated mixing times are shown on the right. Error bars represent the mean ± STD of biological replicates (n = 2 for reactors A and B and polymer reactors; n = 5 for glass reactors). Significant differences are indicated with asterisks (*** *p* < 0.001; **** *p* < 0.0001). (**C**) Maximum infectious virus titers determined with a plaque assay. Black patterns indicate VSE addition. Due to DO control failure in reactor A without VSE at 4 dpi, no fold change is indicated for reactor A.

To eliminate potential effects of 3D-printing and polymer materials, YF-ZIK productions were conducted with and without VSEs in both glass and polymer plain reactors, maintaining identical geometry and mixing times (Figure 9B). As expected, there was no significant difference in VCCs, culture viability, glucose uptake, lactate production, cell-specific growth rates, or virus titers between the glass and 3D-printed polymer plain vessels (Figure 9A–C). Moreover, before infection and/or VSE addition, cell-specific growth rates were consistent across all systems. However, both pDS vessels showed significantly higher lactate production rates (Figure 9B). While some increase in lactate is typical for larger vessels [28], reactor A surprisingly resulted in a higher lactate increase compared to reactor B (2.2-fold compared to 1.8-fold). Following infection without VSEs, both pDS vessels exhibited lower VCCs compared to the 1 L systems, though the VCC and viability trends remained similar (Figure 9A left). The sudden drop in VCC and culture viability observed in reactor A on day 4 pi was somewhat exceptional, and it can be readily explained by a failure of the DO controller. Interestingly, after VSE addition, the cell growth and maximum VCC were identical to the control in both pDS vessels (Figure 9A, right). Notably, the expected reduction in cell growth and maximum VCC, typically observed after VSE addition (Figure 9A), was absent.

Control infections in both pDS vessels led to lower maximum infectious virus titers compared to the 1 L system (Figure 8C). In particular, infections for reactor B resulted in a sixfold reduction in peak infectious virus titers. Due to the DO controller failure in reactor A, infectious virus titers significantly declined at 4 dpi, preventing meaningful comparisons to the control. However, the addition of VSEs led to a substantial increase in peak infectious virus titers in reactor B, reaching up to an 8.2-fold improvement. While the peak titers in VSE-treated pDS vessels remained lower than those observed in the 1 L VSE system, they were similar to the levels of the 1 L control infection (Figure 9C).

## 4. Discussion

Technological advances have significantly improved cell-culture-based vaccine production over the past two decades. However, there remains a critical need for robust manufacturing processes that enable high-yield and scalable viral vaccine production [52]. Ideally, these vaccine production processes would also be transportable to the geographic locations where they are most needed. While process intensification strategies consistently outperform batch-based production across nearly all key domains, including viral titer, facility footprint, cost, and capital expenditure [16,53,54,55,56], their integration into traditional vaccine manufacturing remains challenging and slow. One reason is a continued reliance on adherently proliferating primary and diploid cell substrates and production protocols that are rooted in the 1920s to 1950s [57]. A slow modernization is primarily due to regulatory considerations, reliance on established and paid-off production infrastructure, and concerns that extensive re-evaluation of safety and efficacy in clinical trials may be needed if the production process changes significantly.

Here, we describe a comprehensive method for the optimization of YF-ZIK vaccine production in a fully scalable suspension culture format. Particularly, we demonstrate how commercially available and GMP-ready small molecules (VSEs) can readily be used as a medium additive to increase virus titers without introducing extra complexity to established processes. The results of this study are expected to be applicable to other YFV-based vectors. Importantly, the principle may also be transferable to other live-attenuated and vectored viral vaccines, whereby the type and concentration of VSE may obviously need to be adjusted experimentally. We furthermore describe engineering parameters that impact scaling in different reactor types, with a focus on mixing behaviors that can affect distribution and availability of small effector molecules, such as the VSEs tested here.

### 4.1. Effects of Single- and Multi-VSE Addition on YF-ZIK Titers

VSEs’ modulation of cellular signaling and metabolism may exert pleiotropic effects that may interfere with cell viability. Here, the threshold for final culture viability on 5 dpi was set at 80%, the same level as that observed in the control infection. Final culture viabilities above 75% help to minimize the release of host-cell-derived debris, proteins (including proteases), and DNA. This is operationally important as lowering impurity levels at harvest reduces the complexity of downstream processing and the amount of required endonucleases for the removal of host DNA, which are significant cost factors [55]. For proof of concept, we selected three cell lines for which we previously observed marked differences in maximal virus yields [9]. Despite initial differences in productivity levels of the three tested cell substrates, the addition of VSEs led to an increase in infectious virus titers compared to control infections across all evaluated cell lines. At a small scale, this effect was more pronounced in the low-producing BHK-21 cells, potentially indicating that these cells are poorly permissive, which is due at least in part to the remnant antiviral defenses. There are likely virus-specific dependencies that make BHK-21 less productive for YF-ZIK, since these cells are otherwise highly permissive for a wide range of viruses, including veterinary rabies vaccine strains [58] or genuine YF17D vaccine virus [46]. We subsequently continued the study only with AGE1.CR.pIX and HEK 293 cell lines, as the utility of BHK-21 in human vaccine production has been debated, considering their tendency to produce R-type pseudoparticles [59,60].

Antiviral signatures are functionally and kinetically complex and result from the interaction between a given cell substrate and the virus or vector being produced [61,62]. Consequently, the effectiveness of different VSEs in different cell lines is not unexpected. This could be due to differences in VSE molecular targets, as well as the tolerability of different compounds by a specific cell type. Finally, individual VSE uptake, metabolism, and half-life may differ with the particular culture medium composition (such as pH, metabolites, growth factors, inhibitors, etc.) used for the different cell lines. Interestingly, some combinations (e.g., VS-F + VS-G) exhibited synergies, whereas others were antagonistic (VS-G + VS-B). In our combination studies, dose ranges just below the identified optima for the respective single VSEs were chosen to explore potential synergistic effects while mitigating the risk of excessive cytotoxicity that could impair cell viability and hence viral production. In line with this, we observed reduced tolerability for some compound combinations; for example, the benefits of VS-G + VS-F combinations were abrogated by the addition of one or more additional compounds. Omics studies would be needed to assess the net response of each host cell line to YF-ZIK and the impact of the respective VSEs or combinations thereof given the spatial–temporal complexity, cell- and virus-type dependency of antiviral defense signatures, and interaction between antiviral defense pathways and stress response pathways, which can also be induced by chemical stress (e.g., NRF-2, NF-kB) [63]. While this was outside the scope of the study, the data presented here provide strong rationale to carry out such studies in the future. Appreciating that direct mechanistic evidence is lacking for many critical process parameters, an empirical DoE approach, such as that demonstrated here, allows marked improvements in productivity and justifies the introduction of innovative new modalities such as VSEs. Future mechanistic studies assessing the pro- or antiviral activity of individual VSEs and combinations thereof may use reporter cell lines such as those expressing SEAP (secreted embryonic alkaline phosphatase) or luciferase under promoter elements known to be involved in innate antiviral and stress signaling, such as the NF-kappaB, AP-1, or ISRE (interferon-sensitive) response elements.

### 4.2. Proof of Concept for Translatability and Scale-Up

Direct scale-up over three orders of magnitude is challenging. As shown in our previous study [9], we achieved comparable virus titers in the Ambr15 vessel and 1 L STR cultivations by using tip speed as the sole scale-up parameter. The final culture viabilities on day 5 pi were only marginally affected by the addition of VSEs (70–90%), with obvious benefits for downstream purification of a future drug substance. Moreover, the addition of VSEs to both AGE1.CR.pIX and HEK293-F cells resulted in an over threefold higher infectious YF-ZIK titer compared to previously optimized control infections. Liquid chromatography mass spectrometry measurements showed depletion of VS-C by day 1 pi to below the limit of detection, an observation beneficial for regulatory compliance, as the key quality attributes of the final YF-ZIK product did not change.

### 4.3. Mimicking Large-Scale Production Conditions in Small-Scale 3D-Printed pDS Reactors

One of the most notable differences between the 1 L and pDS vessels was an increased lactate production, a well-known challenge during scale-up that is often used as an indicator of large-scale process behavior [28,64]. The higher lactate concentrations in pDS reactor B could be further explained by a two-phase system simulation that included sparging, free surface simulation, and use of a higher resolution (Supplementary File S2, Figure S9) instead of the initial (faster and less demanding) one-phase simulation that had been employed to generate these pDS reactors (Figure 8B). In a deviation from the simple model, the two-phase simulation reactor B with a reduction in mixing time from 77 s to 19 s more closely resembled a 100–150 L reactor rather that the originally considered 2000 L reactor when taking the total mixing time into account. This highlights the importance of simulating a two-phase system if computationally feasible.

The similar growth performance observed in both pDS reactors following VSE addition, compared to the control, may be attributed to the cytotoxicity of VSE compounds. The stress induced by VSE exposure likely reduces the oxygen consumption rate of the cells. Consequently, the oxygen gradients typically present in large-scale reactors are less likely to impact cell growth, leading to comparable growth patterns across conditions. As anticipated, peak virus titers in the pDS vessels without VSEs were lower than those in the 1 L systems, supporting the hypothesis that transient hypoxia and altered cell metabolism due to prolonged mixing times negatively affect virus production. However, the addition of VSEs led to an increase of up to 8.2-fold in peak virus titers for reactor B (representative of ~100 L reactor). We propose that the previously suggested mechanism, where VSE-induced cellular stress reduces oxygen consumption, thereby minimizing gradients at a larger scale, also explains the restoration of virus production to the levels observed in the 1 L control infection.

### 4.4. Implications of Improved Yields

Bioprocess modeling offers valuable insights into the impacts of increasing viral yields from both a production process and cost perspective. Using a generic cell-based suspension vaccine production model, we modeled the impact of a threefold increase in upstream virus yield at the 2000 L production scale and derived a 61% reduction in manufacturing cost per dose. Furthermore, we explored the impact of reducing the size of the production bioreactor required from 2000 L to 1000 L, enabled by a threefold enhancement in yield. The results of the bioprocess modeling demonstrated that the overall manufacturing cost per dose was decreased by 39%, with the most notable production cost savings being in the materials (−37%) and capital (−8%) cost categories. Additional metrics of interest, water usage (m^3^ per batch) and plastic waste (kg per batch), were decreased by 17% and 6%, respectively. Although this was demonstrated using a generic vaccine model, the results suggest that such increases in yield could enable new opportunities for manufacturing cost reductions, including the reduction in the size of production bioreactors, thereby further reducing process footprints, as well as capital and operational expenditures.

One limitation of this study is that the VSEs are proprietary with undisclosed chemical structures. As a result, a detailed discussion of the impacts of these factors on specific viral replication pathways and cellular defense mechanisms could not be included. However, such a limitation is not unique to our study, as the composition of most commercially available specialty media, feeds, and supplements is usually not fully disclosed. Additionally, even for known compounds (such as sodium butyrate [65]), the effects can be pleotropic, and the actual pathway ultimately responsible for the observed effects can be elucidated only with a differential study of the proteome or transcriptome, an investigation beyond the scope of this investigation. The selected combinations of VSEs investigated during multiple additions were not exhaustive but were rather intended to explore potentially synergistic interactions to enhance virus titers. Future studies should evaluate additional pairings to more comprehensively map the potential of multi-VSE addition. An important property that needs to be addressed during safety-related process development is to ensure that residues of bioactive media additives such as VSEs can be detected with the necessary sensitivity required by regulators and that key quality attributes of the final product remain unchanged. In this context, we demonstrated complete compound depletion by employing very sensitive diagnostics. As such criteria are met, higher yields can be seamlessly integrated into an existing manufacturing setup.

## 5. Conclusions

We demonstrate a significant increase in infectious titers of YF-ZIK at different scales through the addition of single- or multi-component formulations of VSEs in a comprehensive process optimization study. These improved yields were achieved in scalable processes using AGE1.CR.pIX and HEK293 suspension cell lines amenable to human vaccine production. Enhanced productivities were at least threefold in STR and are projected to be potentially eightfold and higher at the 200 L scale and beyond. VSEs may thus serve as a new modality for markedly intensifying live virus vaccine and vector production to match growing global demands.

## Data Availability

Data are available in the Supplementary Material. Additional data are available from the authors upon request.

## Supplementary Materials

The following supporting information can be downloaded at: https://www.mdpi.com/article/10.3390/vaccines13070757/s1. Figure S1 Evaluation of the tolerability of eight VSEs in AGE1.CR.pIX cells; Figure S2 Evaluation of the tolerability of eight VSEs in BHK-21 cells; Figure S3 Evaluation of the tolerability of eight VSEs in HEK293-F cells; Figure S4 Dosing refinement of selected VSEs in AGE1.CR.pIX (A) and HEK293-F (B) cells to increase YF-ZIK titers; Figure S5 Coefficient plot of investigated VSEs; Figure S6 Model statistics; File S1: Residual testing of VSEs by LC-MS; File S2: Two-phase system for verification of pDS reactors.

## Abbreviations

**Abbreviation**	**Meaning**
µ	cell-specific growth rate
AI	artificial intelligence
CCF	reduced face-centered composite design
CFD	computational fluid dynamics
CI	combination index
DO	dissolved oxygen
DoE	design of experiments
FF	full factorial design
LC-MS	liquid chromatography mass spectrometry
MOI	multiplicity of infection
pDS	perfect down-scale system
P/V	volumetric power
PFU	plaque forming units
p.i.	Post-infection
STD	standard deviation
STR	stirred tank bioreactor
VCC	viable cell concentration
VCC_max_	maximum viable cell concentration
VSE	viral sensitizer
wv	working volume
YF17D	yellow fever vaccine strain 17D
YF	yellow fever virus
YF-ZIK	chimeric YF-ZIKprM/E vaccine virus
