# Supplementary material for: Optimization of YF17D-Vectored Zika Vaccine Production by Employing Small-Molecule Viral Sensitizers to Enhance Yields
Source: Vaccines (Basel). 2025 Jul 16;13(7):757. doi: 10.3390/vaccines13070757 (PMC12299442; doi:10.3390/vaccines13070757)
Supplement: Supplementary file 1 [file vaccines-13-00757-s001.zip › vaccines-3707410-supplementary.pdf]

Journal: “Vaccines”

Manuscript Title: “Optimization of YF17D-vectored Zika Vaccine Production employing Small Molecule Viral Sensitizers to Enhance Yields”

Supplementary Material

Table S1: Primers and probes used for real-time RT–qPCR.

Target	Name	Sequence (5`→ 3`)
ZIKV E protein	ZIKV_F	CCGCTGCCCAACACAAG
	ZIKV_R	CCACTAACGTTCTTTTGCAGACAT
	ZIKV_P	6-FAM-AGCCTACCTTGACAAGCAGTCAGACACTCAA-BHQ-1

Primers were obtained from Thermo Scientific and TaqMan-based probes from TIB Molbiol. 373-3

Table S2: Code levels and matrix design of the full factorial DoE. In the Ambr15 system, the experiment numbers were randomized, resulting in random distribution across the 2 units.

**VSE**	**Coding level**		
	**L**	**H**	
VS-G (µM)	10	15	
VS-F (µM)	1	2.5	
VS-B (µM)	3.5	5	
**Experiment number**	**VS-G**	**VS-F**	**VS-B**
1	L	H	-
2	L	L	-
3	H	H	-
4	H	L	-
5	L	-	H
6	L	-	L
7	H	-	H
8	H	-	L
9	L	L	L
10	-	-	-
11	L	H	-
12	L	L	-
13	H	H	-
14	H	L	-
15	L	-	H
16	L	-	L
17	H	-	H
18	H	-	L
19	L	L	L
20	-	-	-

Table S3: Code levels and matrix design of the reduced face-centered composite design DoE. In the Ambr15 system, the experiment numbers were randomized, resulting in random distribution across the 2 units.

VSE	Coding level		
	**-1**	**0**	**1**
VS-G (µM)	3	5.5	8
VS-F (µM)	0.5	1	3
VS-B (µM)	1	3	5
VS-H (µM)	10	15	20

Experiment number	VS-G	VS-F	VS-B	VS-H
1	-1	-1	-1	
2	1	-1	-1	
3	-1	1	-1	
4	1	1	-1	
5	-1	-1	1	
6	1	-1	1	
7	-1	1	1	
8	1	1	1	
9	-1	0	0	
10	1	0	0	
11	0	-1	0	
12	0	1	0	
13	0	0	-1	
14	0	0	1	
15	0	0	0	
16	0	0	0	
17	0	0	0	
18	0	0	0	
19	0	0	0	
20	-1	-1	-1	-1
21	0	0	0	0
22	1	1	1	1
23	-	-	-	-
24	-	-	-	-

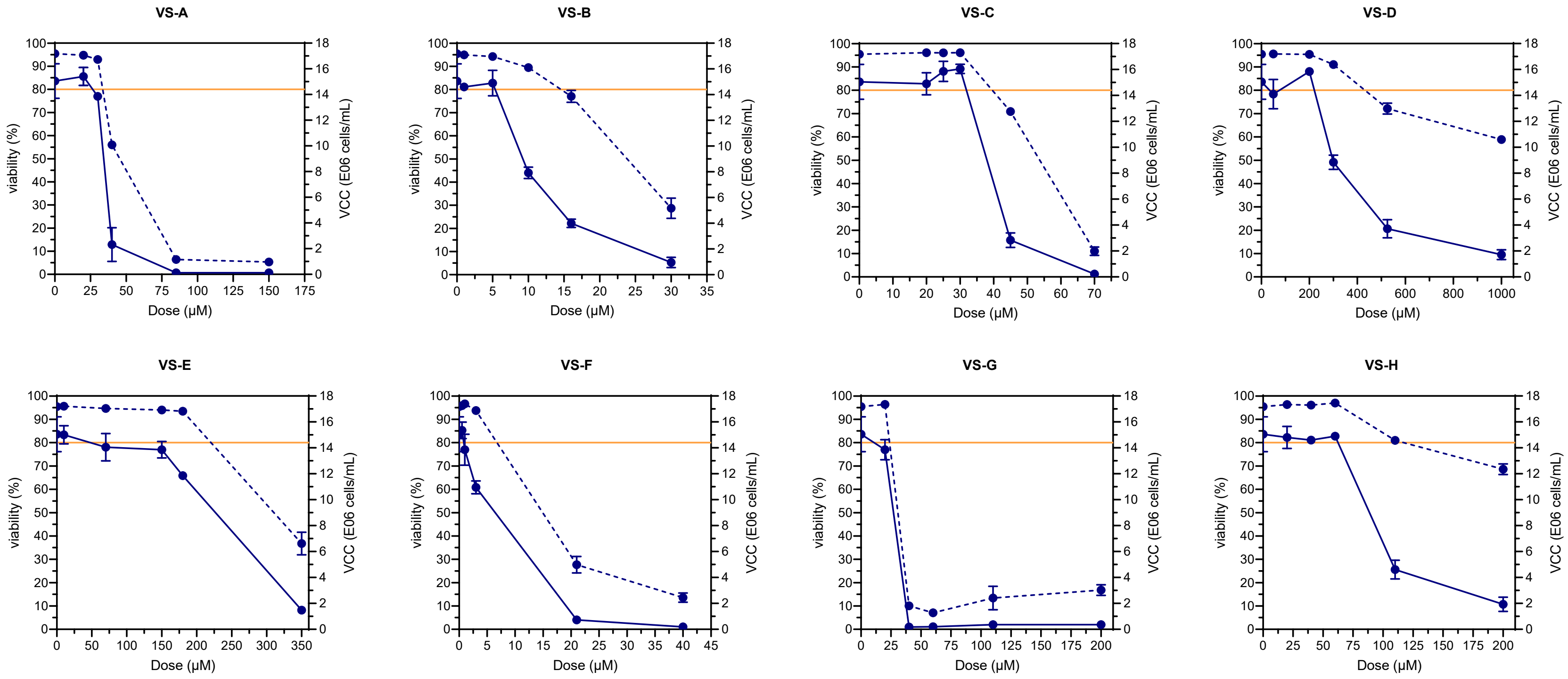

Figure S1 Evaluation of the tolerability of eight VSEs in AGE1.CR.pIX cells. The VSEs used in this study were VS-A, VS-B, VS-C, VS-D, VS-E, VS-F, VS-G, and VS-H. VSEs were added to uninfected cells seeded at $2x10^6$ cells/mL in 50 mL vented falcon spin tubes, harvested 5 days post-addition, and VCC (full lines) and viability (dashed lines) were measured. Error bars represent the mean±STD of biological duplicates. The yellow line represents the pre-defined 80% viability threshold.

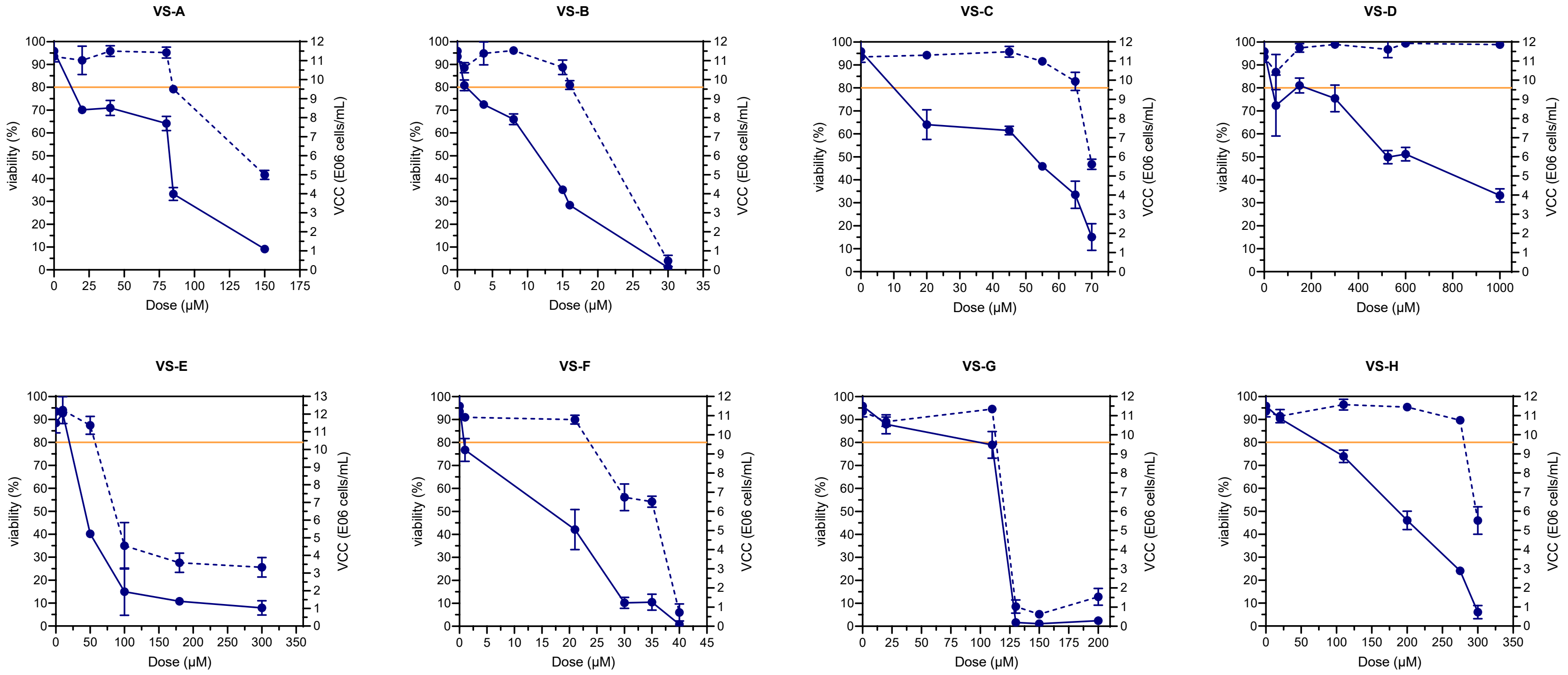

Figure S2 Evaluation of the tolerability of eight VSEs in BHK-21 cells. The VSEs used in this study were VS-A, VS-B, VS-C, VS-D, VS-E, VS-F, VS-G, and VS-H. VSEs were added to uninfected cells seeded at 2x106 cells/mL in 50 mL vented falcon spin tubes, harvested 5 days post-addition, and VCC (full lines) and viability (dashed lines) were measured. Error bars represent the mean±STD of biological duplicates. The yellow line represents the pre-defined 80% viability threshold.

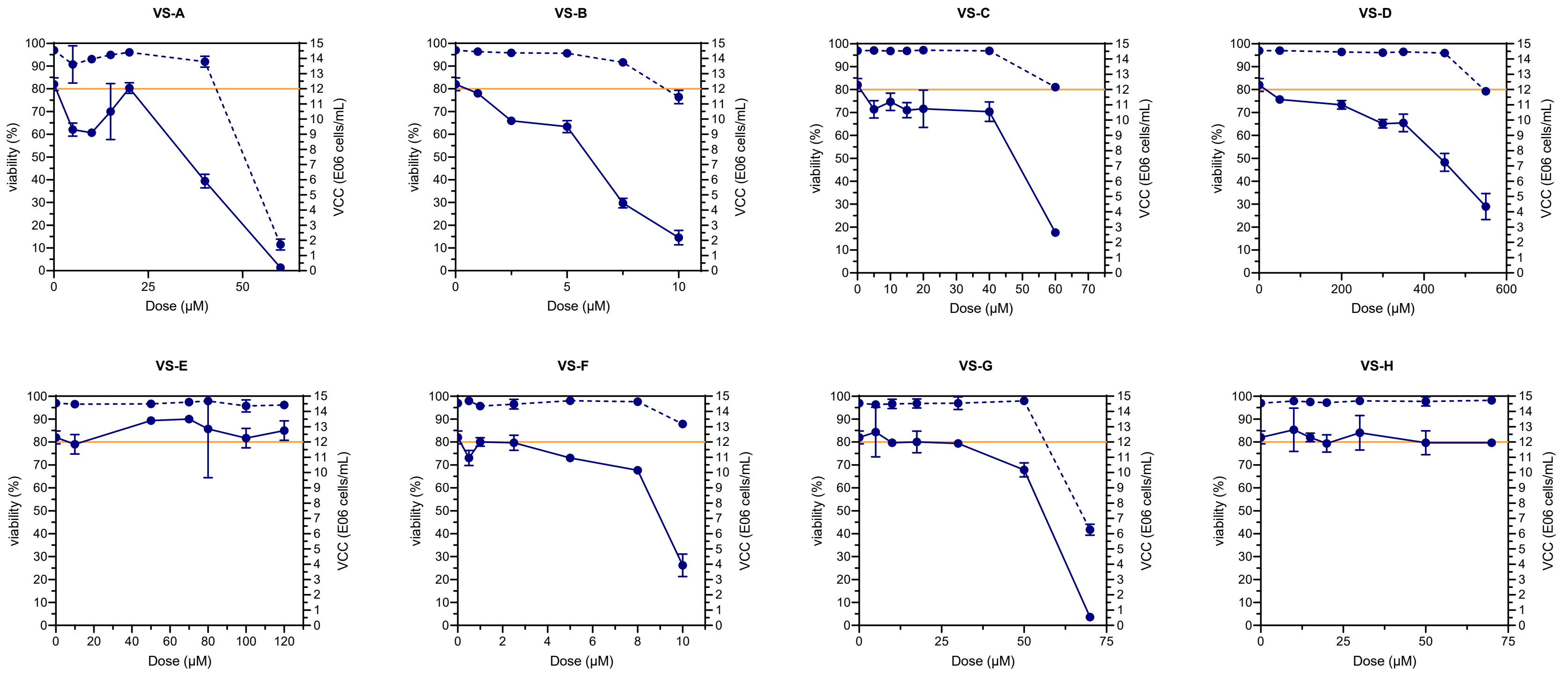

Figure S3 Evaluation of the tolerability of eight VSEs in HEK293-F cells. The VSEs used in this study were VS-A, VS-B, VS-C, VS-D, VS-E, VS-F, VS-G, and VS-H. VSEs were added to uninfected cells seeded at 2x106 cells/mL in 50 mL vented falcon spin tubes, harvested 5 days post-addition, and VCC (full lines) and viability (dashed lines) were measured. Error bars represent the mean±STD of biological duplicates. The yellow line represents the pre-defined 80% viability threshold.

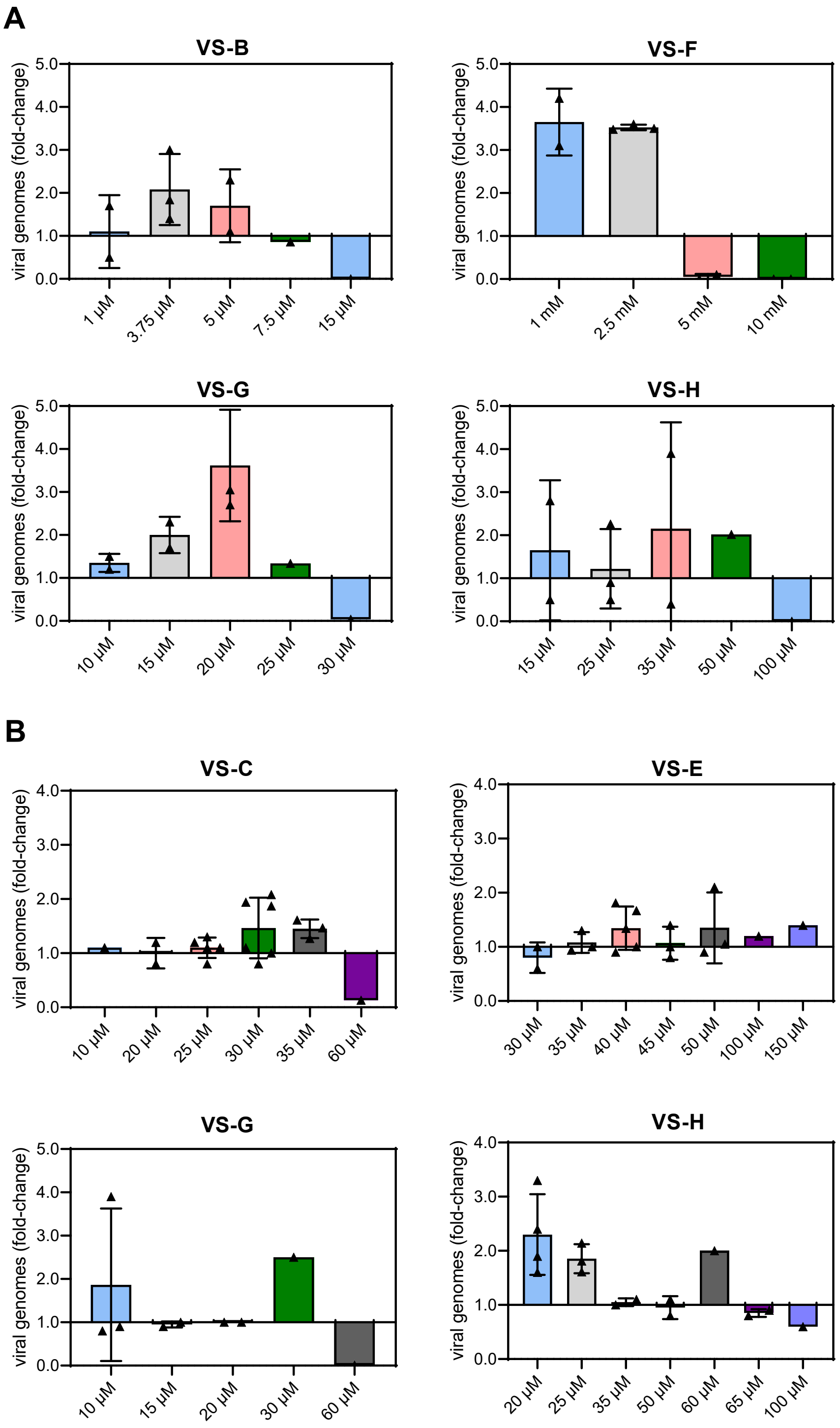

Figure S4 Dosing refinement of selected VSEs in AGE1.CR.pIX (A) and HEK293-F (B) cells to increase YF-ZIK titers. Maximum viral genome titers are shown as fold-changes relative to YF-ZIK producing cells without VSEs. Error bars represent the mean±STD of biological replicates (n=1-6 for VSEs and n=6 for the control).

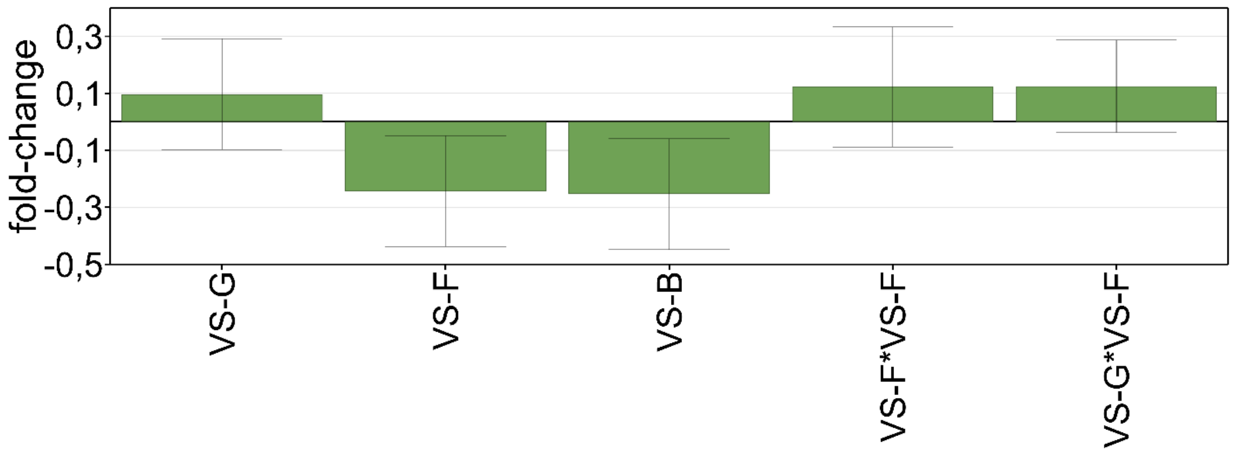

Figure S5 Coefficient plot of investigated VSEs. The influence of VSEs on the target response is shown. Positive and negative coefficients indicate the direction of the effect, while error bars represent the confidence intervals, highlighting the reliability of each parameter's estimate. Insignificant coefficients were excluded unless kept for interaction terms (p-value < 0.05).

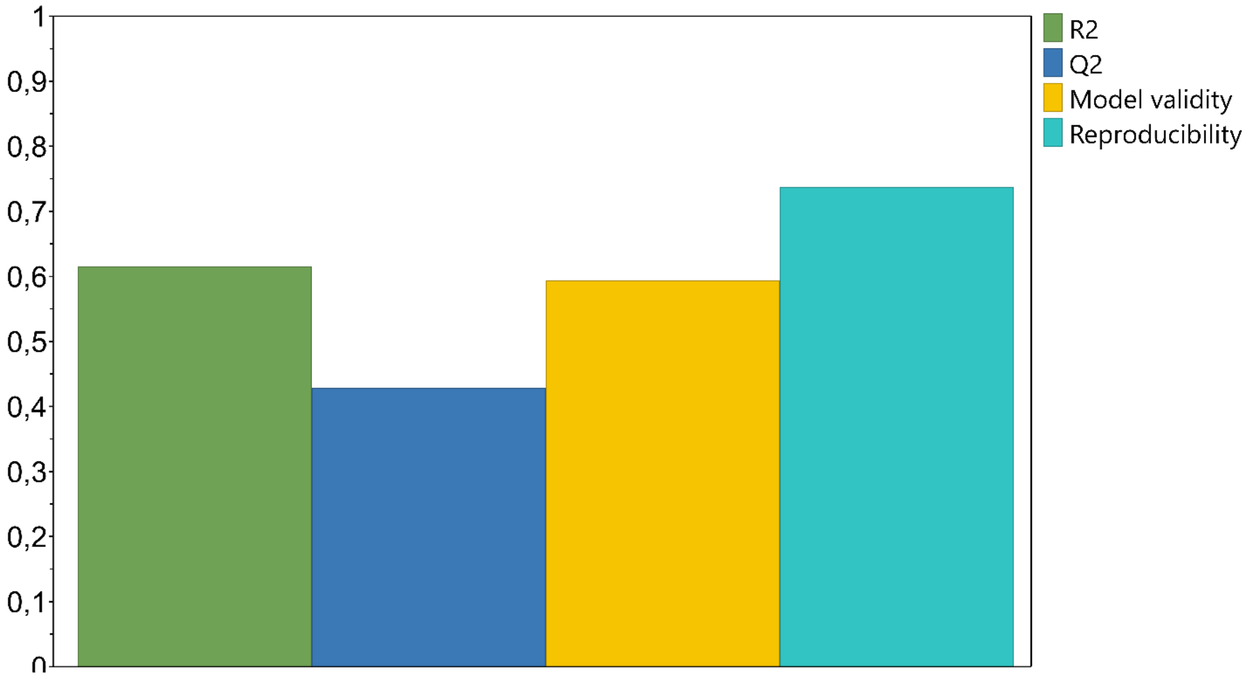

Figure S6 Model statistics. Various statistical metrics were assessed to evaluate the fit and applicability of the model for the reduced CCF design.

Supplementary File S1 - Residual testing of VSEs by LC-MS

Methods

Culture media was thawed at room temperature and filtered through Amicon Ultra Centrifugal 3 kDa molecular weight cut-off filters (Sigma-Aldrich, MO, USA) at 13.8 x *g*, 40 min. Culture media samples from all timepoints of 2 HEK293-F bioreactors containing no VS-C were pooled together and spiked with VS-C at 0.5, 1, 5, 10, 20 and 40 μM to serve as calibrants. Calibrants and bioreactor samples were injected into an Agilent 6546 MS with a 1260 HPLC and an Eclipse Plus C18 (4.6×100 mm, 3.5 μm) column (Agilent Technologies, CA, USA). LiChropur LC-MS grade water and acetonitrile with 0.1% (Sigma-Aldrich, MO, USA) were used as solvents A and B, respectively according to the following gradient program: 0 min 15% B, 0.5 min 15% B, 4 min 99.5% B, 7 min 99.5% B. The flow rate was 0.4 mL/min and a 30 μL injection was used for all samples and calibrants. The detection limit (DL) was assessed by injecting 6 replicates of 0.5 μM VS-C, peak area RSD <20% and signal-to-noise ratio >2000 was recorded for all replicates.

Criteria

Test Method	VSE	Method performance parameters	
LC-MS	VS-C	Quantification Limit (QL)	1.50 μM
		Detection Limit (DL)	0.50 μM

6 replicates of 0.5 μM VS-C in control media were injected to assess the DL. The coefficient of variation was 10.96% for the recorded peak areas for all 6 replicates. No interference was observed in control media without VS-C.

VS-C Peak Area						Average	Standard Deviation
Rep 1	Rep 2	Rep 3	Rep 4	Rep 5	Rep 6		
7738.55	7850.90	7821.15	8603.53	6630.28	6434.51	7513.15	823.60

Results

Sample	day post infection (d)	Result
STR 1: HEK293-F cells with VS-C	0.91	≤ 0.50 µM (BELOW DL)
	1.93	≤ 0.50 µM (BELOW DL)
	2.93	≤ 0.50 µM (BELOW DL)
	3.93	≤ 0.50 µM (BELOW DL)
	4.94	≤ 0.50 µM (BELOW DL)
	5.66	≤ 0.50 µM (BELOW DL)
STR 2: HEK293-F cells with VS-C	0.91	≤ 0.50 µM (BELOW DL)
	1.93	≤ 0.50 µM (BELOW DL)
	2.93	≤ 0.50 µM (BELOW DL)
	3.93	≤ 0.50 µM (BELOW DL)
	4.94	≤ 0.50 µM (BELOW DL)
	5.66	≤ 0.50 µM (BELOW DL)

Controls

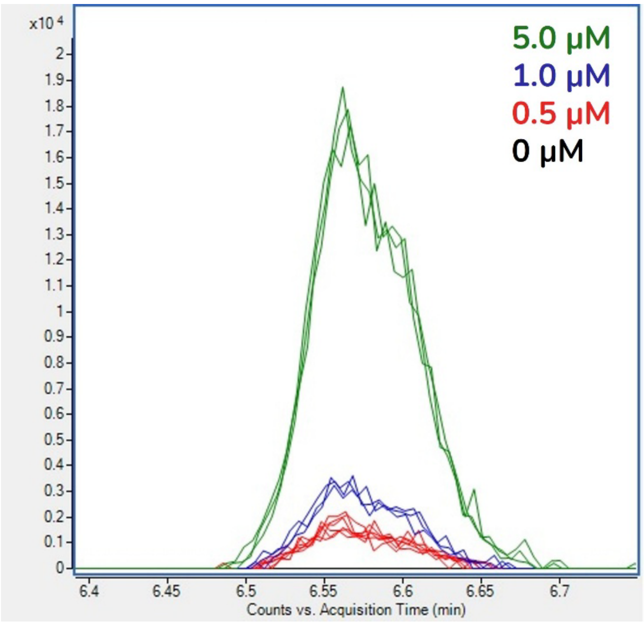

Figure S7. Control media spiked with 0.5, 1.0 and 5.0 µM VS-C. No peak VS-C peak was observed in control media that was not spiked with VS-C, shown as the baseline (black trace).

Calibration Curve

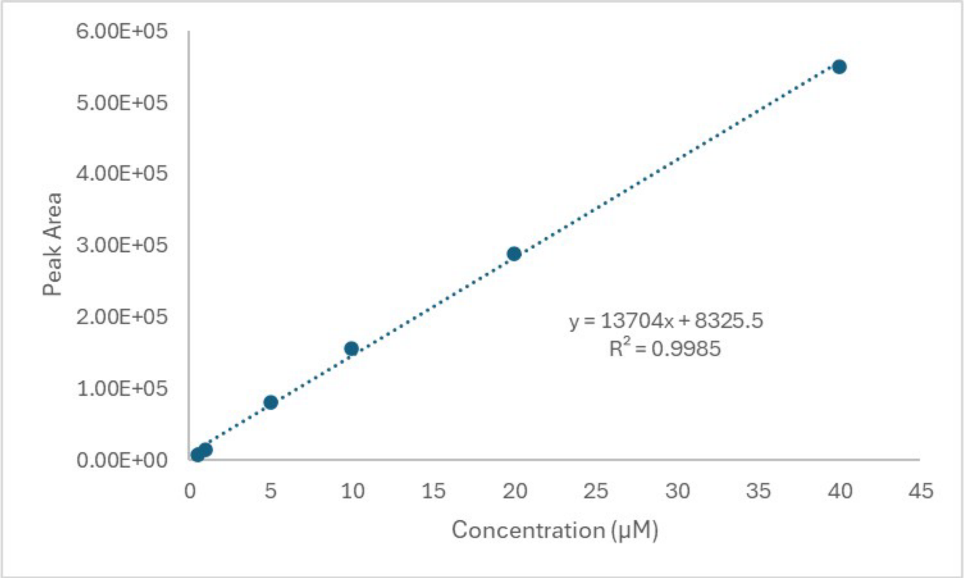

Figure S8. Calibrant peak areas provided a linear response from 0.5 µM to 40 µM (R^2 = 0.9985).

Supplementary File S2 – Two-phase system for verification of pDS reactors

While computational time was saved during the generation of the pDS reactors with a one-phase system simulation, verification of the resulting structured reactors was done using a two-phase system simulation including sparging, free surface simulation and with a higher resolution. Typically, it is expected that mixing times drop when highly structured systems are simulated with higher resolution, and gassing reduces mixing times as well. The simulations were evaluated for mixing curves as described in the materials and method section and the two different pDS reactors showed different behavior in terms of variability of results with and without two-phase system. While reactor A modelled for 200 L showed only small differences when changing from a one-phase system to a two-phase system (mixing time dropped from 39 to 33 seconds with some changes to the shape of the mixing curve), reactor B modelled for 2000 L showed significant differences with a drop in mixing time from 77 seconds to 19 seconds, more closely resembling an actual 100-150 L reactor, than a 2000 L. We suspect that the difference sizes of structures used in both modes (with finer structure for 2000 L) is one main driving factor why one structure is more susceptible to switching to a two-phase system. Looking at this data, one of the reactors in this study (200 L) is displaying a close mixing behavior to what it’s supposed to model, also in a two-phase system, but one (2000 L) more closely resembles a 100 L reactor when being gassed. This also highlights the importance of simulating two-phase system if computationally feasible, already for generating the small-scale reactors.

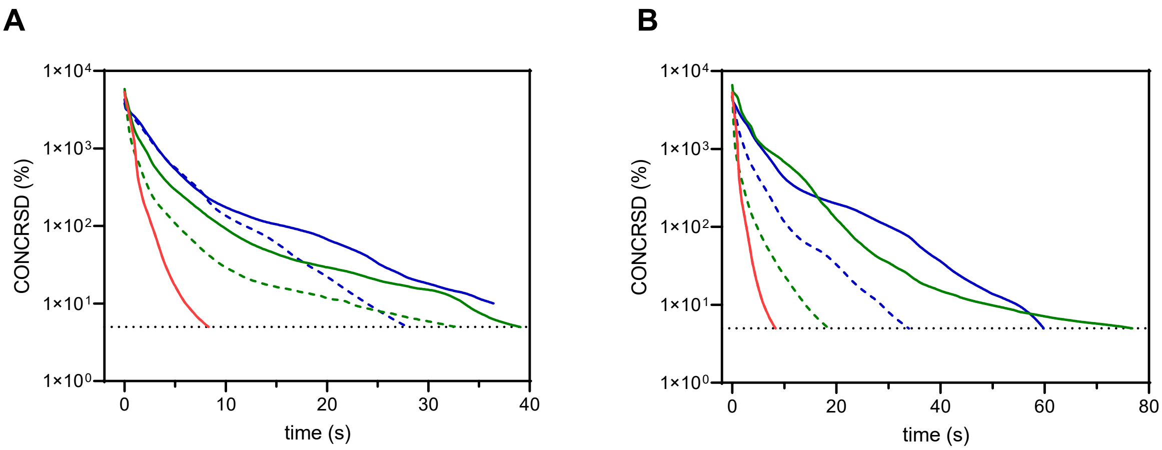

Figure S9 Difference between gassed and non-gassed conditions for large scale, small scale and pDS. A) shows the difference in gassed (dashed lines) and non-gassed (full lines) conditions for the small scale (red), 200 L reference (blue) and 200 L pDS scale (green). **B)** shows the difference between gassed (dashed lines) and non-gassed (full lines) conditions for the small scale (red), 2000 L reference (blue) and 2000 L pDS scale (green).
